# Astragaloside IV in hepatocellular carcinoma: repolarizing macrophages to reprogram the tumor microenvironment

**DOI:** 10.3389/fphar.2026.1931925

**Published:** 2026-09-25

**Authors:** Peng Ma, Xin-da Lu, Jian-li Han, Jia-jia Du

**Affiliations:** 1 Shanxi Bethune Hospital, Shanxi Academy of Medical Sciences, Third Hospital of Shanxi Medical University, Tongji Shanxi Hospital, Taiyuan, China; 2 Third Hospital of Shanxi Medical University, Shanxi Bethune Hospital, Shanxi Academy of Medical Sciences, Tongji Shanxi Hospital, Taiyuan, China

**Keywords:** Astragaloside IV, hepatocellular carcinoma, macrophage polarization, tumor microenvironment, tumor-associated macrophages

## Abstract

The establishment of an immunosuppressive tumor microenvironment (TME) represents a central mechanism underlying the failure of conventional therapies and resistance to immunotherapy in hepatocellular carcinoma (HCC). Tumor-associated macrophages (TAMs), as the most abundant immune cell population within this microenvironment, play a pivotal role in establishing immunosuppression, promoting angiogenesis, and driving therapeutic resistance through their polarization toward the M2 phenotype. Astragaloside IV (AS-IV), the principal bioactive constituent of the traditional Chinese medicine Astragalus membranaceus, has recently been recognized for its remarkable immunomodulatory properties. This review systematically summarizes recent advances in understanding the multi-dimensional mechanisms through which AS-IV modulates M2 macrophage polarization. At the signaling pathway level, AS-IV undermines the molecular basis of macrophage polarization by inhibiting signal transducer and activator of transcription 3/6 (STAT3/STAT6) phosphorylation and modulating phosphatidylinositol 3-kinase/protein kinase B/mammalian target of rapamycin (PI3K/Akt/mTOR) and AMP-activated protein kinase (AMPK) signaling cascades. At the metabolic level, it influences the metabolic reprogramming of macrophages, thereby altering their functional phenotypes. At the epigenetic level, it may interfere with processes such as histone modifications, thereby attenuating the “memory” of M2 polarization. This multi-layered regulation promotes the reprogramming of TAMs from a pro-tumorigenic M2 phenotype toward an anti-tumor M1-like phenotype, thereby systematically remodeling the HCC immune microenvironment—restoring effector T cell function, reducing immunosuppressive cell infiltration, inhibiting angiogenesis, and ameliorating the fibrotic barrier. This review aims to provide a systematic framework for understanding the immunomodulatory mechanisms of AS-IV and offer a theoretical foundation for the development of novel natural product-based immunotherapeutic strategies for HCC.

## 1 Introduction

Hepatocellular carcinoma (HCC), a highly prevalent malignant tumor associated with substantial morbidity and mortality worldwide, represents a major global public health challenge. According to recent global cancer statistics, HCC accounts for approximately 800,000 deaths annually, ranking as the third leading cause of cancer-related mortality globally (Butt et al., 2026). Despite remarkable advances in HCC treatment strategies over the past decade—including refinements in surgical techniques, optimization of local ablation modalities, and the emergence of systemic therapies represented by immune checkpoint inhibitors (ICIs)—the overall prognosis of patients remains dismal, with a 5-year survival rate hovering around 18% (Dopazo et al., 2024). The core of this therapeutic dilemma lies in the high heterogeneity and complex biological characteristics of HCC, particularly the profoundly immunosuppressive microenvironment it establishes.

The HCC tumor microenvironment (TME) constitutes a dynamic ecosystem comprising diverse cell types and soluble mediators. Within this system, the functional state of immune cells dictates the tumor’s immune response profile and therapeutic responsiveness. Among these, tumor-associated macrophages (TAMs)—the most abundant population of tumor-infiltrating immune cells, accounting for approximately 30%–50% of the total immune infiltrate—exert a critical and dual regulatory role in governing tumor immune responses (Tang et al., 2025). Under normal physiological conditions, macrophages possess the capacity to recognize and eliminate abnormal cells; however, under the continuous shaping of the TME, they predominantly polarize toward the M2 phenotype, alternatively termed the “alternatively activated” phenotype. These M2-type TAMs not only lose their anti-tumor functions but also actively establish and maintain an immunosuppressive state by secreting immunosuppressive factors such as interleukin-10 (IL-10) and transforming growth factor-β (TGF-β), expressing immune checkpoint molecules such as programmed death ligand-1 (PD-L1), and depleting arginine in the microenvironment through metabolic reprogramming—thereby promoting tumor growth, angiogenesis, invasion, and metastasis (Xu et al., 2025). Numerous clinical studies have confirmed that high-density infiltration of M2-type macrophages in tumor tissue is significantly associated with poor prognosis, increased risk of early recurrence, and shorter overall survival in HCC patients, rendering them an attractive therapeutic target (Liu F. et al., 2025).

Nevertheless, the clinical translation of TAM-targeted strategies faces substantial challenges. Traditional approaches, such as macrophage depletion or blockade of their recruitment, often yield limited efficacy and may compromise physiological macrophage functions (Mantovani et al., 2022). Consequently, the development of strategies capable of precisely reprogramming TAM polarization states—driving their transition from the pro-tumorigenic M2 phenotype toward the anti-tumor M1-like phenotype—has emerged as a cutting-edge Frontier in tumor immunotherapy research in recent years (Cheng et al., 2022). This “re-education” rather than “depletion” strategy aims to restore immune surveillance function within the TME while avoiding systemic immune toxicity.

Against this backdrop, natural compounds derived from traditional medicine have regained considerable attention owing to their multi-target actions, relatively favorable safety profiles, and long history of clinical application (Su et al., 2025). Astragaloside IV (AS-IV), one of the principal bioactive constituents extracted from the dried roots of Astragalus membranaceus, belongs to the cycloartane-type tetracyclic triterpenoid saponins. Previous pharmacological research has primarily focused on its anti-inflammatory, antioxidant, cardioprotective, and direct anti-proliferative effects on tumor cells (Liang et al., 2023). However, with advances in immunometabolism and tumor immunology research, accumulating evidence has revealed that AS-IV possesses significant immunomodulatory functions, particularly the ability to precisely modulate the macrophage polarization process. In the context of HCC, AS-IV has demonstrated unique potential in ameliorating the immunosuppressive microenvironment by regulating TAM function (Gao X. et al., 2025).

Despite its promising immunomodulatory properties, the clinical translation of AS-IV is constrained by its suboptimal pharmacokinetic profile. Following oral administration, AS-IV exhibits low systemic bioavailability (approximately 7.4% in rats) due to poor aqueous solubility and limited intestinal permeability (Zhang et al., 2006). Furthermore, its distribution to liver tissue, while detectable, may not achieve therapeutically effective concentrations without dose escalation or formulation optimization. These exposure limitations represent a critical translational barrier that must be addressed through advanced delivery strategies.

This review aims to systematically elucidate and comprehensively explore the molecular mechanisms, functional effects, and clinical translation prospects of AS-IV in remodeling the immunosuppressive microenvironment of HCC by regulating TAM polarization. Through this systematic analysis, we aim to provide a solid theoretical foundation and novel insights for the development of AS-IV-based immunotherapeutic strategies for HCC.

## 2 Biological characteristics and functions of M2-type macrophages in the HCC immune microenvironment

### 2.1 Polarization drivers and heterogeneity

The HCC microenvironment drives monocyte recruitment to tumor sites and their subsequent polarization toward the M2 phenotype through the secretion of a diverse array of chemokines and cytokines, forming a complex signaling network (Figure 1). Among these, the chemokine C-C motif chemokine ligand 2 (CCL2) and its receptor C-C chemokine receptor 2 (CCR2) play a central role in monocyte recruitment (Avila and Berasain, 2019). CCL2 secreted by tumor cells and stromal cells establishes a concentration gradient that guides peripheral blood monocytes across the endothelial barrier and into tumor tissue. Additionally, CXCL12 (stromal cell-derived factor-1α) also participates in this process through its receptor C-X-C chemokine receptor 4 (CXCR4) (Lian et al., 2024). Once within the TME, these monocytes undergo polarization under the continuous stimulation of specific cytokines.

**FIGURE 1:**
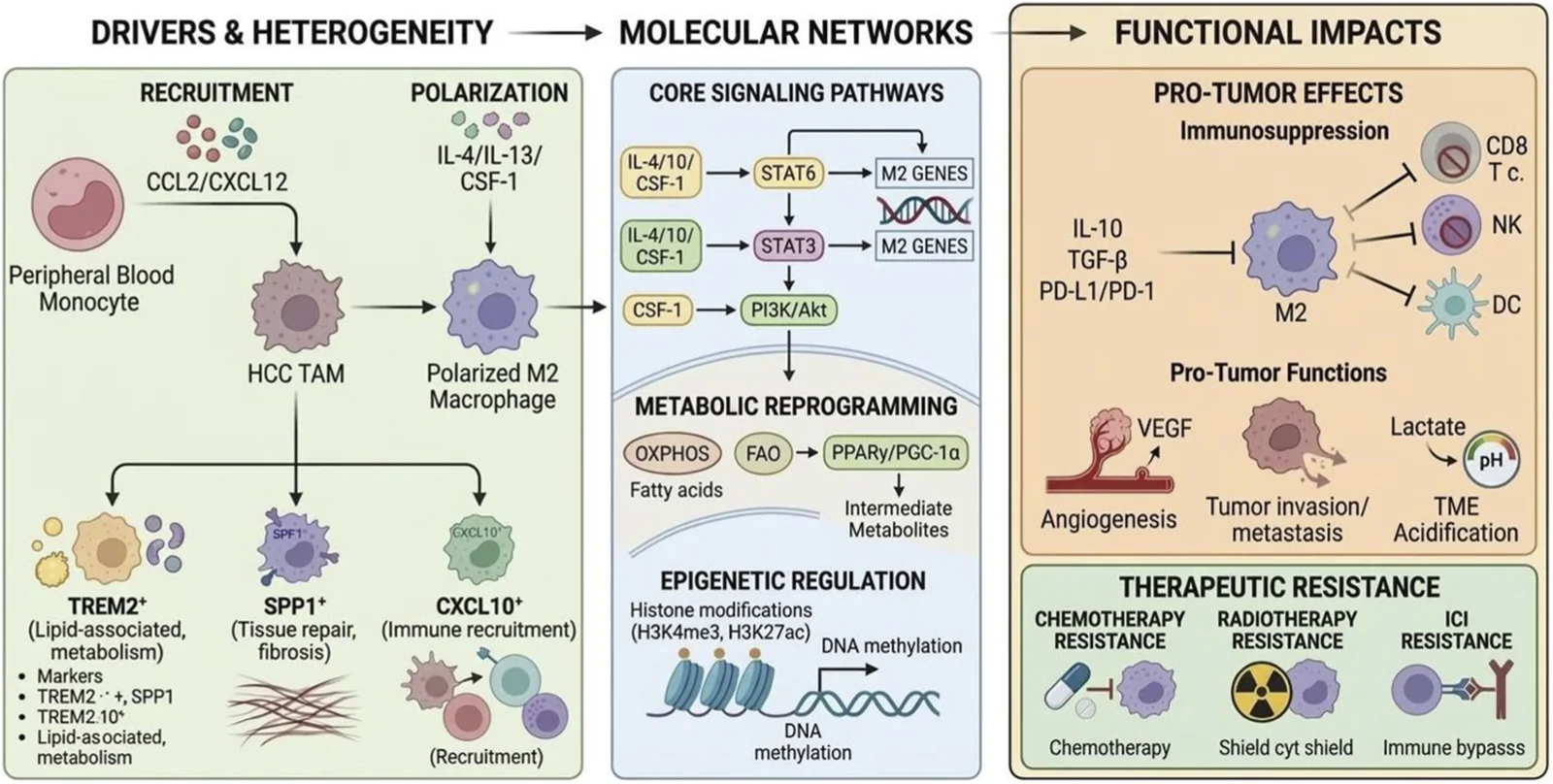
Multifaceted pro-tumorigenic roles of M2-type tumor-associated macrophages in the hepatocellular carcinoma microenvironment. M2-type TAMs exert diverse pro-tumorigenic effects in the HCC microenvironment through multiple mechanisms: (1) establishing an immunosuppressive state by secreting inhibitory cytokines and expressing immune checkpoint molecules; (2) promoting angiogenesis via the release of pro-angiogenic factors and matrix metalloproteinases; (3) driving tumor invasion and metastasis by inducing epithelial-mesenchymal transition; (4) maintaining cancer stem cell niches; and (5) contributing to therapeutic resistance.

Key cytokines driving M2 polarization include colony-stimulating factor-1 (CSF-1), interleukin-4 (IL-4), IL-10, and interleukin-13 (IL-13). CSF-1 not only promotes monocyte survival and proliferation but also induces the expression of M2-associated genes through activation of downstream signaling pathways such as phosphatidylinositol 3-kinase/protein kinase B (PI3K/Akt) (Cao and Wang, 2024). IL-4 and IL-13 are classical M2 polarization inducers; they upregulate the expression of M2 markers such as arginase-1 (Arg1) and mannose receptor through activation of the Janus kinase 1/signal transducer and activator of transcription 6 (JAK1/STAT6) signaling axis (Yang Y. et al., 2026). IL-10, conversely, induces macrophages to produce more IL-10 via activation of the STAT3 pathway, forming a self-reinforcing immunosuppressive loop (Li et al., 2025). These signaling pathways do not operate independently but rather constitute a cross-talking network that collectively determines the final macrophage phenotype.

In recent years, the application of single-cell RNA sequencing technology has profoundly advanced our understanding of TAM heterogeneity. Studies have demonstrated that TAMs in HCC do not represent a homogeneous M2 population but rather a continuous spectrum encompassing multiple functional subsets. For instance, Zheng et al. identified at least three major macrophage subsets in HCC via single-cell analysis: 1) TREM2^+^ lipid-associated macrophages, which highly express lipid metabolism-related genes and may be associated with tumor lipid accumulation and immunosuppression; 2) SPP1^+^ macrophages, which highly express osteopontin and are implicated in tissue repair, fibrosis, and immune regulation; and 3) CXCL10^+^ macrophages, which express higher levels of chemokines and may possess enhanced immune cell recruitment potential (Zheng et al., 2017). This spatiotemporal heterogeneity suggests that targeted therapies may require precise design tailored to distinct tumor stages and individual patient characteristics.

### 2.2 HCC-specific features of the macrophage microenvironment

While many aspects of M2-TAM biology are shared across solid tumors, the hepatic microenvironment confers several unique features that distinguish HCC-associated macrophages from those in other malignancies. These HCC-specific characteristics have important implications for the design and interpretation of macrophage-targeted therapies, including AS-IV-based strategies.

#### 2.2.1 Chronic inflammation–fibrosis–carcinogenesis axis

A defining feature of HCC is that most cases arise on a background of chronic hepatitis, fibrosis, and cirrhosis. Activated hepatic stellate cells (HSCs) secrete abundant TGF-β, IL-6, and ECM proteins that promote and maintain M2-like TAM polarization (Baglieri et al., 2019). Unlike most other solid tumors, the fibrotic liver thus provides a pre-existing immunosuppressive niche established long before tumor initiation. This has two major implications: the macrophage-suppressive network is embedded in a chronically sustained wound-healing program, and therapeutic strategies aimed solely at TAMs may be insufficient because HSCs and the fibrotic stroma continuously replenish M2-polarizing signals. For AS-IV, this suggests that maximal efficacy may require concurrent targeting of HSC activation and fibrotic stroma remodeling. The reported anti-fibrotic activity of AS-IV in hepatic fibrosis models therefore represents an important, yet underexplored, component of its anti-TAM activity in HCC (Liu et al., 2020).

#### 2.2.2 Gut–liver axis

HCC patients frequently exhibit gut microbiota dysbiosis, increased intestinal permeability, and elevated portal vein levels of lipopolysaccharide (LPS) (Tripathi et al., 2018). The liver is uniquely exposed to gut-derived microbial products via the portal circulation, and LPS signaling through TLR4/NF-κB in hepatic macrophages drives a distinctive activation state that reinforces the M2-like phenotype. This sustained low-grade endotoxemia contributes to the immunosuppressive microenvironment and may blunt immunotherapy efficacy. Preliminary evidence indicates that AS-IV can protect the intestinal barrier and modulate gut microbiota composition (Zhang et al., 2020); however, whether these gut-directed effects contribute to its anti-TAM activity in HCC is unknown. Investigating whether AS-IV reduces portal LPS levels and thereby dampens TLR4-driven macrophage polarization represents an important future direction.

#### 2.2.3 Abnormal bile acid metabolism

Bile acids are dysregulated in chronic liver disease and HCC, functioning not only as detergents but also as signaling molecules through FXR and TGR5 (Fiorucci and Distrutti, 2015). TGR5 is expressed on macrophages, and its activation by bile acids suppresses pro-inflammatory cytokine production while promoting an M2-like, tissue-protective phenotype. Sustained TGR5 signaling may therefore reinforce the immunosuppressive macrophage compartment in HCC. Whether AS-IV modulates bile acid composition or directly interferes with TGR5/FXR signaling in TAMs has not been investigated and constitutes a significant knowledge gap. This is particularly relevant because bile acid profiles vary considerably depending on HCC etiology (viral, metabolic, alcohol-related), potentially creating patient-specific differences in macrophage polarization pressure.

#### 2.2.4 Kupffer cells versus monocyte-derived TAMs

A critical feature of the hepatic macrophage compartment is its dual developmental origin. Liver-resident Kupffer cells (KCs), originating from yolk-sac precursors, are radio-resistant, self-renewing, and express high levels of CD163 and TIM-4. In contrast, monocyte-derived TAMs (Mo-TAMs) recruited via CCL2/CCR2 signaling tend to express CCR2, CD11b, and higher levels of PD-L1 (Scott et al., 2016). Functionally, Mo-TAMs are generally considered the more immunosuppressive and pro-tumorigenic subset, whereas KCs may retain tissue-protective and phagocytic functions. Whether AS-IV differentially regulates these two populations is currently unknown. This distinction carries direct therapeutic relevance: CD206/CD163-targeted nanocarriers—commonly proposed for macrophage-directed drug delivery—may preferentially deliver AS-IV to Kupffer cells rather than to the more pathogenic Mo-TAMs, potentially limiting efficacy or causing off-target effects. Conversely, if AS-IV exerts stronger effects on Mo-TAMs, CCR2-targeted delivery systems may be more appropriate. Resolving this question requires single-cell-resolution studies comparing the transcriptomic and functional responses of KCs and Mo-TAMs to AS-IV treatment in HCC models.

### 2.3 Multi-dimensionality of core immunosuppressive mechanisms

M2-type TAMs efficiently establish and maintain the tumor immunosuppressive microenvironment through a repertoire of multi-dimensional, synergistic mechanisms, which can be broadly categorized into direct suppression, metabolic interference, and physical barriers.

#### 2.3.1 Direct suppressive mechanisms

Cytokine-mediated suppression represents the most canonical immunosuppressive mechanism employed by M2-type TAMs. The high levels of IL-10 they secrete can inhibit dendritic cell (DC) maturation and antigen presentation function, downregulate the expression of major histocompatibility complex class II (MHC-II) molecules and co-stimulatory molecules, and thereby impair T cell activation (Shiri et al., 2024). Concurrently, IL-10 can directly suppress the capacity of CD8^+^ cytotoxic T cells and T helper 1 (Th1) cells to produce effector cytokines such as interferon-γ (IFN-γ). TGF-β is another potent immunosuppressive factor; it not only inhibits the proliferation and cytotoxicity of CD8^+^ T cells and natural killer (NK) cells but also serves as a key driver of naïve CD4^+^ T cell differentiation into regulatory T cells (Tregs) (Peng et al., 2022). Tregs further secrete IL-10 and TGF-β within the TME, thereby amplifying the immunosuppressive effect.

The expression of immune checkpoint molecules constitutes a direct pathway through which M2-type TAMs mediate T cell exhaustion. These cells highly express PD-L1 on their surface; when PD-L1 binds to PD-1 on T cells, it transmits inhibitory signals, leading to T cell proliferation arrest, diminished effector function, and ultimately exhaustion or apoptosis (Pu and Ji, 2022). In addition to PD-L1, co-inhibitory molecules such as B7-H4 (VTCN1) are also expressed on M2-type macrophages, further suppressing T cell function through interactions with as-yet-unidentified receptors on T cells (Kumar et al., 2025).

#### 2.3.2 Metabolic interference mechanisms

Metabolic competition within the TME represents an immune escape mechanism that has attracted considerable attention in recent years, with M2-type macrophages playing a significant role therein. Their highly expressed arginase-1 (Arg1) hydrolyzes L-arginine in the microenvironment into ornithine and urea (Zhu et al., 2025). L-arginine is essential for T cell activation and proliferation, serving as a critical substrate for metabolic reprogramming and nitric oxide synthesis. Arginine depletion directly leads to downregulation of the T cell receptor ζ chain, arresting T cells in the G0-G1 phase and preventing effective proliferation and cytotoxic function. The product of Arg1, ornithine, serves as a precursor for polyamine synthesis, and polyamines have also been shown to exert immunosuppressive effects.

Furthermore, the glycolytic activity of M2-type macrophages produces large quantities of lactate, which—together with lactate produced by tumor cells—contributes to TME acidification (pH values as low as 6.0–6.5) (Gu et al., 2024). This acidic environment exerts multiple negative effects on immune cell function: it inhibits the cytotoxicity and cytokine production of T cells and NK cells; impairs the antigen processing and presentation capabilities of dendritic cells (DCs); and may promote the stability and suppressive function of Tregs. Lactate itself can also act as a signaling molecule, inhibiting the immune activation function of antigen-presenting cells through its receptor GPR81.

#### 2.3.3 Physical barriers and cell-cell interactions

In addition to secreted factors, M2-type TAMs also construct physical and functional barriers through interactions with other stromal cells. They engage in close crosstalk with cancer-associated fibroblasts (CAFs): CAFs secrete factors such as CCL2 and CSF-1 to recruit and polarize macrophages, while macrophages activate CAFs by secreting factors such as platelet-derived growth factor (PDGF), promoting extracellular matrix deposition and fibrosis (Sun et al., 2025). This dense fibrotic stroma not only constitutes a physical barrier that impedes cytotoxic T cell infiltration but also its components (such as collagen and hyaluronic acid) can themselves transmit inhibitory signals.

### 2.4 Synergistic effects in promoting tumor progression

The pro-tumor functions of M2-type TAMs extend far beyond immunosuppression; they participate in virtually every aspect of tumor progression and form an intimate symbiotic relationship with tumor cells.

#### 2.4.1 Angiogenesis and lymphangiogenesis

Tumor growth and metastasis are highly dependent on the formation of new blood vessels. M2-type TAMs serve as critical “helper cells” for tumor angiogenesis (Hou et al., 2024). They secrete a variety of pro-angiogenic factors—including vascular endothelial growth factor (VEGF), fibroblast growth factor-2 (FGF-2), PDGF, and TGF-β—directly stimulating endothelial cell proliferation, migration, and lumen formation. Spatially, these macrophages often aggregate around tumor blood vessels, forming the so-called “perivascular” phenomenon, supporting vascular stability and function through direct cell contact or paracrine signaling. Additionally, they secrete matrix metalloproteinases (such as MMP-2 and MMP-9) and ephrins, degrading vascular basement membranes and remodeling the extracellular matrix to pave the way for angiogenesis. Similarly, they also promote tumor lymphangiogenesis by secreting factors such as VEGF-C, creating conditions for lymphatic metastasis.

#### 2.4.2 Invasion and metastasis

Tumor invasion and metastasis represent a multi-step, complex process in which M2-type macrophages play a driving role. They directly promote tumor cell proliferation by secreting mitogens such as epidermal growth factor (EGF) (Hu et al., 2024). More importantly, they can induce epithelial-mesenchymal transition (EMT) in tumor cells by secreting various cytokines (such as TGF-β, tumor necrosis factor-α (TNF-α), and interleukin-6 (IL-6)) and proteases. EMT enables tumor cells to lose intercellular junctions and polarity, acquiring the migratory and invasive capabilities of mesenchymal cells—a critical step in distant metastasis. Macrophage-derived proteases (such as MMPs and cathepsins) degrade the extracellular matrix and basement membrane, opening channels for tumor cell invasion. Some studies have even observed that macrophages form fusion cells with tumor cells or exchange substances through tunneling nanotubes, which may directly transfer metastatic potential.

#### 2.4.3 Maintenance of cancer stem cell niches

Cancer stem cells (CSCs)—a small subpopulation of tumor cells with self-renewal, multi-differentiation, and drug resistance capabilities—are considered the root of tumor recurrence and metastasis. M2-type TAMs maintain and support CSCs by creating specialized microenvironmental niches (Chen et al., 2022). For instance, IL-6 secreted by them can activate the STAT3 signaling pathway in CSCs, a critical pathway for maintaining stemness. Macrophages also influence CSC metabolism and drug resistance by secreting mediators such as prostaglandin E2 (PGE2) and nitric oxide (NO). In certain tumors, a bidirectional positive feedback loop exists between macrophages and CSCs: CSCs secrete factors to recruit and polarize macrophages, while polarized macrophages in turn maintain CSC properties.

#### 2.4.4 Therapeutic resistance

M2-type TAMs are associated with multiple forms of therapeutic resistance. In chemotherapy, they can secrete cytokines to protect tumor cells from apoptosis or activate survival pathways such as nuclear factor κ-light-chain-enhancer of activated B cells (NF-κB) and STAT3 (Kzhyshkowska et al., 2024). In radiotherapy, macrophages help tumor cells repair DNA damage and resist radiation-induced cell death by secreting mediators such as nitric oxide synthase (NOS) and PGE2. Particularly importantly, in ICI therapy, M2-type macrophages are recognized as one of the key factors underlying primary or acquired resistance. They not only directly inhibit T cells through molecules such as PD-L1 but also establish compensatory immunosuppressive networks by recruiting other immunosuppressive cells such as Tregs and myeloid-derived suppressor cells (MDSCs). Therefore, targeting M2-type macrophages holds promise as a breakthrough for overcoming existing therapeutic resistance and improving treatment efficacy.

### 2.5 Complexity of the molecular regulatory network

The acquisition and maintenance of the M2 phenotype depend on a complex intracellular molecular regulatory network involving precise regulation at multiple levels—signal transduction, metabolic reprogramming, and epigenetic modifications.

#### 2.5.1 Core signaling pathways

##### 2.5.1.1 JAK/STAT pathway

This is the core pathway that responds to extracellular signals and initiates M2 gene transcription. IL-4/IL-13 activate JAK1/JAK3 through receptors, which in turn phosphorylate STAT6. Phosphorylated STAT6 forms dimers, translocates to the nucleus, and binds to the promoter regions of M2-associated genes (such as Arg1, Retnla, and Mrc1), driving their expression (Fortelny et al., 2024). IL-10 and CSF-1, on the other hand, primarily exert their polarization effects by activating STAT3. STAT3 not only directly promotes M2 gene expression but also reinforces the M2 phenotype by inducing the expression of transcription factors such as IRF4 and inhibiting IRF5.

##### 2.5.1.2 PI3K/Akt/mTOR pathway

This pathway integrates growth factor, cytokine, and nutrient signals and serves as a central regulator of cell growth, survival, and metabolism. In M2 polarization, PI3K/Akt activation promotes the M2 phenotype through multiple mechanisms, including activation of downstream mammalian target of rapamycin complex 1 (mTORC1)—a major driver of protein synthesis and metabolic reprogramming (Xue et al., 2024). Inhibition of mTORC1 can block IL-4-induced M2 polarization, suggesting its critical role in phenotypic regulation.

##### 2.5.1.3 AMPK pathway

AMPK is the cellular energy sensor. Unlike the pro-inflammatory effects of AMPK activation in M1 polarization, the activity and function of AMPK in the context of M2 polarization are more complex. Some studies suggest that AMPK activation may indirectly influence M2 polarization by inhibiting mTORC1 or regulating mitochondrial function (Cui et al., 2023). AMPK is closely linked to cellular metabolic status and represents a key node connecting metabolism and immunity.

#### 2.5.2 Metabolic reprogramming

Macrophage polarization is accompanied by profound metabolic remodeling; metabolic status is both a consequence of polarization and, conversely, influences and stabilizes the cellular phenotype. M1 macrophages primarily rely on glycolysis for rapid ATP production—even under aerobic conditions (the Warburg effect)—accompanied by the pentose phosphate pathway and a truncated tricarboxylic acid (TCA) cycle (Pan et al., 2022). In contrast, M2 macrophages are metabolically characterized by enhanced oxidative phosphorylation (OXPHOS) and fatty acid oxidation (FAO). This metabolic preference enables M2 macrophages to utilize long-chain fatty acids more efficiently as an energy source, supporting their tissue repair and immunoregulatory functions. This process is regulated by peroxisome proliferator-activated receptor γ (PPARγ) and its coactivator peroxisome proliferator-activated receptor γ coactivator 1α (PGC-1α). PPARγ directly promotes the expression of genes related to fatty acid uptake and oxidation and acts synergistically with STAT6 to reinforce the M2 transcriptional program.

#### 2.5.3 Epigenetic regulation

Epigenetic modifications provide “memory” for macrophage polarization, enabling cells to maintain specific functional states even after the initial stimulus has disappeared. Histone modifications represent an important aspect of this. For instance, M2-associated gene loci are often accompanied by increases in activating histone marks such as H3K4me3 (trimethylation) and H3K27ac (acetylation), as well as decreases in the repressive mark H3K27me3 (Daskalaki et al., 2025). These modifications alter chromatin accessibility, facilitating transcription factor binding. The activities of histone deacetylases (HDACs) and histone methyltransferases/demethylases are regulated by polarization signals. DNA methylation also plays a role in polarization; M2 polarization may lead to demethylation of specific gene promoter regions, thereby permitting their sustained expression. Interestingly, intermediate products of cellular metabolism—such as acetyl-CoA, α-ketoglutarate, and S-adenosylmethionine—serve precisely as substrates or cofactors for histone modifications and DNA methylation, thus tightly linking cellular metabolism to epigenetic regulation.

Notably, this metabolic-epigenetic coupling is central to the sustained maintenance of the M2 phenotype. Within the TME, specific metabolites act as substrates or cofactors for epigenetic enzymes, establishing a self-reinforcing loop. For instance, α-ketoglutarate (α-KG), a TCA cycle intermediate, functions as an obligatory co-substrate for ten-eleven translocation (TET) DNA demethylases and JmjC-domain histone demethylases, both of which promote M2-associated gene programs (Loenarz and Schofield, 2011). Conversely, the M2 metabolic signature—characterized by elevated fatty acid oxidation (FAO) and glutamine utilization—drives the intracellular accumulation of acetyl-CoA and S-adenosylmethionine (SAM), which respectively fuel histone acetyltransferases (HATs) and histone/DNA methyltransferases (Wellen et al., 2009). These enzymatic activities reinforce an open chromatin conformation at M2 marker loci (e.g., Arg1, Mrc1) and maintain their transcriptional competence across cell divisions. AS-IV, by disrupting FAO and altering the intracellular pools of acetyl-CoA and α-KG, may deprive these epigenetic enzymes of their essential substrates, thereby destabilizing the chromatin landscape that sustains M2 polarization memory.

## 3 Molecular characteristics and pharmacological activities of AS-IV

### 3.1 Chemical structure and pharmacokinetic features

AS-IV is a cycloartane-type tetracyclic triterpenoid saponin extracted and isolated from the dried roots of the Chinese medicinal herb Astragalus membranaceus. Its chemical name is 3-O-β-D-xylopyranosyl-6-O-β-D-glucopyranosylcycloastragenol, with a molecular formula of C_41_H_68_O_14_ and a molecular weight of 784.97 (Zhang et al., 2020). Its chemical structure comprises a hydrophobic cycloartane triterpenoid aglycone and two hydrophilic glycosyl moieties (one xylose and one glucose) linked via glycosidic bonds. This amphipathic structure enables it to interact with the phospholipid bilayer of cell membranes and may modulate membrane protein function, serving as a critical structural basis for its multi-target pharmacological activities. The multiple hydroxyl groups and glycosyl moieties in its structure also confer relatively good aqueous solubility, although oral absorption remains challenging.

Pharmacokinetic studies indicate that AS-IV is absorbed slowly and incompletely following oral administration, with a low absolute bioavailability (approximately 2.2%–5.3%) (Yang G. et al., 2025). This is primarily attributed to its relatively large molecular weight, low lipophilicity, instability in the gastrointestinal tract, and potential first-pass effects. Following absorption, AS-IV is widely distributed throughout the body but primarily accumulates in the liver, kidneys, and lungs—consistent with the liver being its primary metabolic organ and its potential hepatoprotective and renoprotective targeting properties. In plasma, AS-IV exhibits a high protein binding rate, which influences its free drug concentration and volume of distribution.

The metabolic transformation of AS-IV *in vivo* is relatively complex, primarily involving phase I metabolic reactions mediated by the hepatic cytochrome P450 enzyme system (particularly CYP3A4 and CYP2C9), including hydroxylation and deglycosylation, which yield multiple metabolites (Li et al., 2023). Among these, deglycosylation products (such as cycloastragenol) may still retain partial biological activity. AS-IV and its metabolites are ultimately excreted primarily via renal elimination in urine, with small amounts eliminated through biliary and fecal pathways. Its elimination half-life varies across studies but is generally relatively short, suggesting that frequent administration or sustained-release formulations may be required to maintain stable plasma concentrations.

To improve the bioavailability and targeting of AS-IV, the development of nano-delivery systems has emerged as a prominent research focus in recent years. For instance, encapsulating it in liposomes, polymeric nanoparticles (such as poly(lactic-co-glycolic acid) nanoparticles), solid lipid nanoparticles, or mesoporous silica nanoparticles can significantly enhance its aqueous solubility, protect it from degradation by gastrointestinal enzymes, and enable passive accumulation at tumor sites through the enhanced permeability and retention (EPR) effect (Kong et al., 2024). More advanced strategies involve the development of actively targeted nanocarriers, such as modifying nanoparticle surfaces with mannose (targeting macrophage mannose receptors), RGD peptides (targeting tumor vascular endothelial integrin αvβ3), or folic acid (targeting tumor cells with high folate receptor expression) to achieve precise delivery to the TME or specific cell types (Liu Y. et al., 2025). Furthermore, stimuli-responsive drug delivery systems (such as pH-sensitive, enzyme-sensitive, or reactive oxygen species-sensitive carriers) can leverage the unique characteristics of the TME to achieve controlled drug release at target sites, further enhancing therapeutic efficacy and reducing systemic toxicity (Yang J. et al., 2025).

Despite promising preclinical results, nano-delivery systems for AS-IV remain at the preclinical stage, and several bottlenecks hinder clinical translation. First, carrier immunogenicity poses a significant concern: many nanocarriers can activate complement or trigger hypersensitivity upon repeated dosing, potentially worsening immune-related toxicities in HCC patients. Second, the metabolic fate and long-term toxicity of nanocarrier degradation products remain poorly characterized, particularly under impaired hepatic clearance. Third, large-scale manufacturing with reproducible physicochemical properties presents substantial technical and regulatory hurdles. Moreover, commonly used targeting ligands (e.g., mannose, galactose) are not truly TAM-specific—their receptors (CD206, etc.) are also expressed on normal tissue macrophages (e.g., Kupffer cells, alveolar macrophages) and even some non-macrophage cells. This off-target accumulation risks reprogramming homeostatic macrophages and disrupting immune surveillance. Future development should prioritize identifying more specific TAM markers, designing dual-targeting or stimuli-responsive systems, and rigorously evaluating off-target effects in relevant preclinical models.

#### 3.1.1 Pharmacokinetic challenges in populations with hepatic impairment

The clinical translation of AS-IV is further complicated by the fact that many HCC patients have underlying cirrhosis or hepatic insufficiency, which substantially alters drug-metabolizing enzyme activity. CYP3A4 and CYP2C9, key enzymes in AS-IV metabolism, are often downregulated in cirrhotic livers, potentially reducing first-pass clearance and raising systemic exposure. Concurrently, reduced hepatic blood flow, portosystemic shunting, and hypoalbuminemia can further alter drug distribution and free fraction, making the pharmacokinetic profile of AS-IV highly unpredictable in this population. Even modest drug accumulation in patients with compromised hepatic function may exacerbate toxicities or disturb liver-resident macrophage homeostasis. These considerations highlight the need to evaluate AS-IV pharmacokinetics and safety margins in preclinical models of hepatic impairment and to develop delivery systems that minimize reliance on hepatic metabolism.

### 3.2 Multi-level pharmacological activities

AS-IV exhibits a broad spectrum of pharmacological activities; its actions are not limited to a single target but rather exert effects through a multi-dimensional, multi-level mechanistic network, which confers unique advantages in the treatment of complex diseases such as cancer.

#### 3.2.1 Direct anti-tumor effects

Numerous *in vitro* and *in vivo* studies have confirmed that AS-IV exerts direct growth-inhibitory and pro-apoptotic effects on various tumor cell types. In HCC cell lines (such as HepG2, Huh7, and SMMC-7721), AS-IV treatment inhibits cell proliferation in a dose- and time-dependent manner (Wu et al., 2024). The mechanisms underlying its induction of apoptosis involve: 1) Mitochondrial pathway: downregulating the expression of anti-apoptotic proteins Bcl-2 and Bcl-xL, upregulating the expression of pro-apoptotic proteins Bax and Bak, leading to decreased mitochondrial membrane potential, cytochrome c release, and subsequent activation of the caspase-9 and caspase-3 cascade; 2) Death receptor pathway: under certain conditions, it may upregulate the expression of Fas/FasL and activate caspase-8; 3) Endoplasmic reticulum stress pathway: inducing the expression of endoplasmic reticulum stress markers such as GRP78 and CHOP, and activating caspase-12.

AS-IV can also induce cell cycle arrest, causing cells to become arrested in G0/G1 phase or G2/M phase. The mechanism is related to upregulating the expression of cyclin-dependent kinase inhibitors p21 and p27 while simultaneously downregulating the expression of cyclins (such as cyclin D1 and cyclin E) and cyclin-dependent kinases (such as CDK4 and CDK2) (Lee et al., 2021). Additionally, AS-IV significantly inhibits the invasion and metastatic capabilities of HCC cells. This is closely associated with its downregulation of the expression and activity of MMPs (particularly MMP-2 and MMP-9), upregulation of the expression of their endogenous inhibitors tissue inhibitor of metalloproteinases-1 (TIMP-1) and tissue inhibitor of metalloproteinases-2 (TIMP-2), and reversal of the EMT process (upregulating E-cadherin and downregulating N-cadherin, vimentin, and transcription factors such as Snail) (Yang Y. et al., 2025).

#### 3.2.2 Basal immunomodulatory activities

AS-IV exerts a bidirectional regulatory effect on the immune system—that is, it enhances immunity when immune function is suppressed and inhibits immunity when inflammation is excessive—consistent with the traditional Chinese medicine theory of “strengthening the righteous and dispelling the pathogenic.”

##### 3.2.2.1 Regulation of T cells

AS-IV can promote the differentiation of naïve CD4^+^ T cells toward Th1 cells, manifested by upregulated T-bet expression and increased IFN-γ secretion, while simultaneously inhibiting differentiation toward T helper 2 (Th2) and regulatory T (Treg) cells. This contributes to establishing a cellular immune response favorable for anti-tumor immunity (Yang J. et al., 2026).

##### 3.2.2.2 Regulation of DCs

As the most professional antigen-presenting cells, the maturation state of DCs determines the efficiency of T cell activation. AS-IV can promote DC maturation, upregulate the expression of surface MHC-II molecules and co-stimulatory molecules (CD80, CD86, CD40), and enhance their IL-12 secretion capacity, thereby more effectively activating naïve T cells (Guo et al., 2022).

##### 3.2.2.3 Anti-inflammatory effects

In various acute and chronic inflammation models, AS-IV demonstrates significant anti-inflammatory effects. The mechanisms involve inhibiting the activation of the nuclear factor κB (NF-κB) signaling pathway, reducing the production of pro-inflammatory cytokines (such as TNF-α, IL-1β, and IL-6), and suppressing the expression of inducible nitric oxide synthase (iNOS) and cyclooxygenase-2 (COX-2) (Yang et al., 2022).

#### 3.2.3 Tumor immune microenvironment-specific regulation

This represents the most cutting-edge and translationally promising direction in AS-IV anti-tumor research. Unlike traditional chemotherapeutic agents that primarily target tumor cells, AS-IV can act on multiple stromal cells within the TME—particularly immune cells—thereby systematically altering the tumor ecosystem.

##### 3.2.3.1 Reprogramming TAMs

As will be elaborated in subsequent sections of this review, AS-IV can effectively inhibit macrophage polarization toward the M2 phenotype and may promote their shift toward an M1-like phenotype. This represents one of the core mechanisms by which it reverses the immunosuppressive microenvironment (Min et al., 2022).

##### 3.2.3.2 Regulation of MDSCs

MDSCs represent another important class of immunosuppressive cells. Preliminary studies suggest that AS-IV may reduce the recruitment and expansion of MDSCs in tumors and attenuate their ability to suppress T cell function by inhibiting such as the STAT3 pathway (Guo L. et al., 2025).

##### 3.2.3.3 Improving tumor vascular function

AS-IV possesses vascular protective effects. In the tumor context, it may reduce abnormal angiogenesis by inhibiting VEGF secretion from M2-type TAMs and potentially promote vascular “normalization,” improving tumor perfusion and hypoxia, thereby facilitating immune cell infiltration and drug delivery (He et al., 2025).

##### 3.2.3.4 Regulation of CAFs

CAFs are the main component of the tumor stroma. Studies suggest that AS-IV may inhibit CAF activation by interfering with signaling pathways such as TGF-β/Smad, reducing excessive extracellular matrix deposition, and thereby lowering the physical barrier and ameliorating immunosuppression (Liu et al., 2023).

## 4 Molecular mechanisms of AS-IV in regulating macrophage polarization

### 4.1 Precise intervention at the signaling pathway level

The regulation of macrophage polarization by AS-IV is first manifested in its precise intervention in key signaling pathways, which serve as bridges connecting extracellular stimuli with the conversion of intracellular gene expression programs (Table 1; Figure 2).

**TABLE 1:** Multi-level molecular mechanisms of Astragaloside IV in regulating M2-to-M1 macrophage polarization.

Regulatory level	Targeted molecules/pathways	Mechanisms of action	Functional consequences	References
Signaling pathway	JAK/STAT3/STAT6 axis	Inhibits phosphorylation of STAT3 (Tyr705) and STAT6; Enhances SOCS1/3 expression; Blocks nuclear translocation of p-STAT3/6	Downregulates M2 markers (Arg1, CD206, CD163); Reduces secretion of IL-10 and TGF-β	(Wang et al., 2025; Yang and Wang, 2019)
	PI3K/Akt/mTOR cascade	Inhibits phosphorylation of PI3K p110/p85, Akt (Thr308/Ser473), mTOR, p70S6K and 4E-BP1	Attenuates protein synthesis and metabolic reprogramming required for M2 phenotype maintenance	(Zhang et al., 2024)
	AMPK signaling	Induces phosphorylation and activation of AMPK; Inhibits downstream mTORC1; Regulates PGC-1α-mediated mitochondrial biogenesis	Shifts cellular energy metabolism from anabolic to catabolic states	(Kuang et al., 2025)
Metabolic reprogramming	Fatty acid oxidation (FAO)	Downregulates PPARγ/PGC-1α expression; Reduces CD36-mediated fatty acid uptake; Inhibits CPT1 activity	Disrupts the primary energy source of M2 macrophages	(Luo et al., 2021)
	Arginine metabolism	Downregulates Arg1 expression via STAT6 inhibition; Alters arginine metabolic flux	Reduces production of immunosuppressive polyamines; Increases availability of arginine for iNOS pathway	(Yue et al., 2020)
	Oxidative phosphorylation (OXPHOS)	Modulates mitochondrial membrane potential; Regulates electron transport chain complex activity	Impairs energy production efficiency in M2 macrophages	(Zhang et al., 2025a)
Epigenetic regulation	Histone modifications	Alters intracellular acetyl-CoA and α-ketoglutarate levels; Activates SIRT1 (NAD^+^-dependent HDAC)	Changes chromatin accessibility at M2-associated gene loci; Attenuates M2 polarization “memory”	(Zhou et al., 2017; Qin et al., 2023)
Cellular functional changes	Secretory profile	Reduces secretion of M2-associated factors (IL-10, TGF-β, CCL17, CCL22); May increase M1-associated cytokines (IL-12, TNF-α)	Creates a pro-inflammatory, anti-tumor microenvironment	(Zhou et al., 2024; Hu et al., 2025)
	Phagocytic and antigen-presenting capacity	Enhances phagocytosis of tumor cells; Upregulates MHC-II and co-stimulatory molecules (CD80, CD86)	Improves tumor antigen presentation to T cells	(Wang et al., 2026)

**FIGURE 2:**
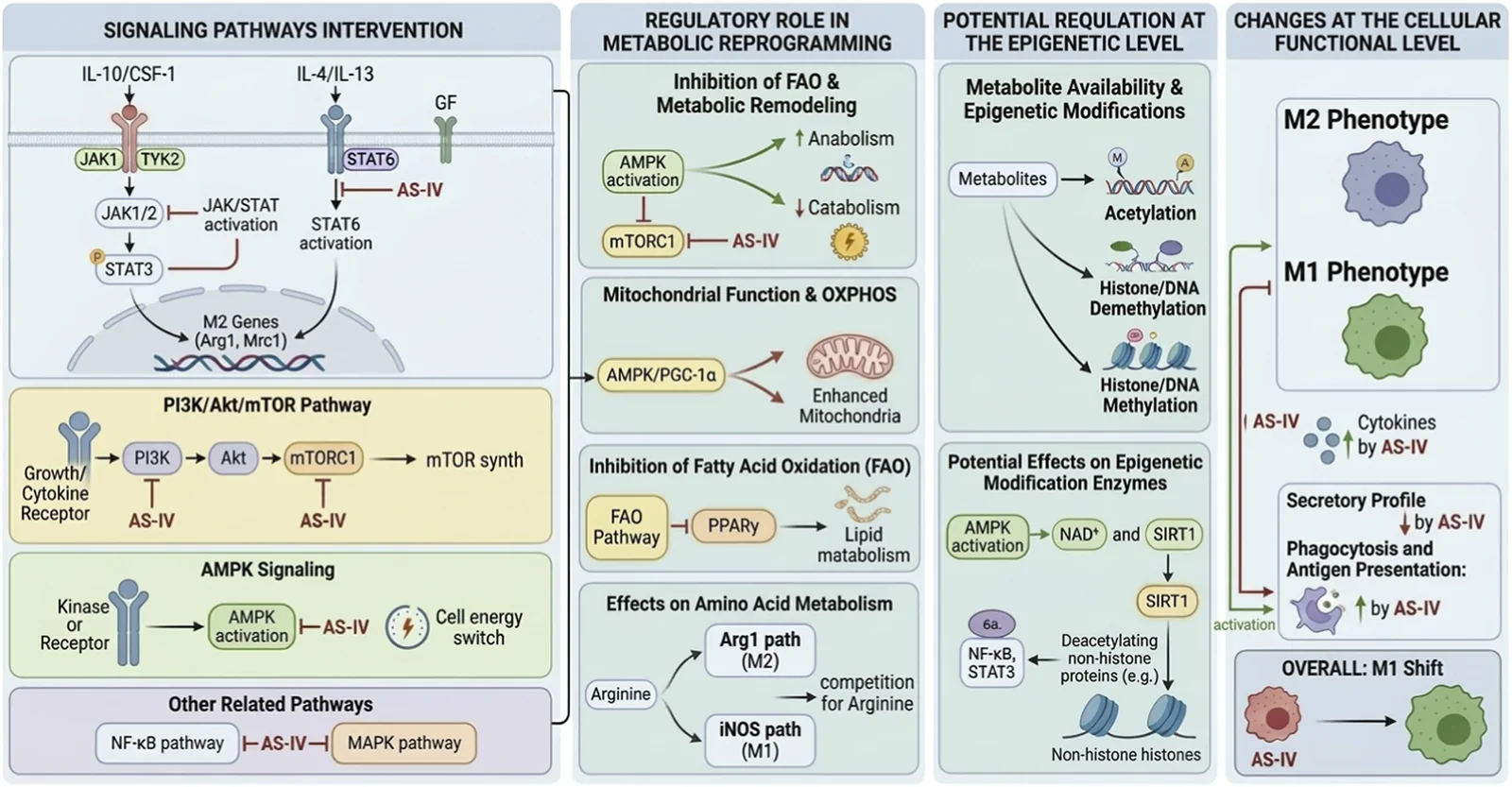
Multi-dimensional molecular mechanisms of astragaloside IV in regulating macrophage polarization. Astragaloside IV modulates macrophage polarization through three interconnected levels of regulation. At the signaling pathway level, it inhibits JAK/STAT3/6 and PI3K/Akt/mTOR pathways while activating AMPK. At the metabolic level, it reprograms cellular energy metabolism by suppressing oxidative phosphorylation and fatty acid oxidation. At the epigenetic level, it influences histone modifications through altering metabolite availability. These synergistic effects collectively drive the reprogramming of macrophages from a pro-tumorigenic M2 phenotype toward an anti-tumor M1-like phenotype.

#### 4.1.1 Regulation of the JAK/STAT pathway

The JAK/STAT pathway is the central pathway for cytokine signal transduction, and the sustained activation of STAT3 and STAT6 is critical for driving M2-type gene expression. Studies have demonstrated that AS-IV can effectively interfere with this pathway.

##### 4.1.1.1 Inhibition of STAT3 activation

In macrophage M2 polarization models induced by IL-10, CSF-1, or tumor cell conditioned medium, AS-IV pretreatment significantly reduces the phosphorylation level of STAT3 at the Tyr705 site and inhibits its nuclear translocation (Wang et al., 2025). Phosphorylated STAT3 dimers, upon entering the nucleus, act as transcription factors that directly bind to the promoter regions of M2 marker genes such as Arg1 and Mrc1 (CD206). AS-IV intervention blocks this process, leading to decreased transcription levels of downstream M2-associated genes. Mechanistically, AS-IV may: 1) Inhibit the activity of upstream JAK family kinases (such as JAK1 and JAK2); 2) Enhance the activity of protein tyrosine phosphatases (such as SHP-1 and SHP-2), leading to STAT3 dephosphorylation; 3) Upregulate the expression of suppressor of cytokine signaling (SOCS) family proteins (such as SOCS1 and SOCS3). SOCS proteins exert negative feedback inhibition on JAK/STAT signaling by competitively binding to JAK or receptors and promoting protein ubiquitination and degradation.

##### 4.1.1.2 Inhibition of STAT6 activation

For the more classical M2a-type polarization induced by IL-4 or IL-13, AS-IV similarly demonstrates inhibitory effects. It can reduce STAT6 phosphorylation, thereby interfering with the IL-4Rα/JAK/STAT6 signaling axis. This leads to significant downregulation of STAT6-dependent M2a signature genes (such as Arg1, Fizz1, and Ym1) (Yang and Wang, 2019). The inhibition of STAT6 by AS-IV may be related to its regulation of upstream signaling molecules or its effect on the DNA-binding capacity of STAT6.

#### 4.1.2 Regulation of the PI3K/Akt/mTOR pathway

The PI3K/Akt/mTOR pathway integrates growth factor, cytokine, and nutrient signals and serves as a core regulator of cellular metabolism, growth, survival, and autophagy; it is also closely associated with macrophage polarization.

##### 4.1.2.1 Multi-node inhibition

AS-IV can inhibit the activation of multiple key nodes in this pathway. Studies have found that it reduces the phosphorylation of the PI3K catalytic subunit p110 and regulatory subunit p85, decreasing their membrane recruitment and activity (Zhang et al., 2024). Consequently, the phosphorylation of its downstream key kinase Akt at the Thr308 and Ser473 sites is also inhibited. Akt inactivation further leads to reduced activity of its downstream effector mTORC1, manifested by decreased phosphorylation levels of mTOR and its downstream targets p70S6K and 4E-BP1.

##### 4.1.2.2 Functional implications

Inhibition of the PI3K/Akt/mTOR pathway exerts multiple effects on macrophage polarization. First, mTORC1 is a major driver of protein synthesis and metabolic reprogramming; its inhibition alters cellular metabolic status, which in turn determines the functional phenotype of macrophages. Second, this pathway engages in crosstalk with STAT3 and other molecules; inhibiting PI3K/Akt may indirectly affect STAT3 activity. Finally, this pathway also regulates cellular autophagy, and the autophagic process participates in regulating macrophage inflammatory responses and polarization.

#### 4.1.3 Regulation of AMPK signaling

AMPK is the cellular energy sensor; it is activated during nutrient deprivation, promoting catabolism to generate energy while inhibiting anabolism. The activation state of AMPK is complexly associated with macrophage polarization.

##### 4.1.3.1 Activation of AMPK

Multiple studies have demonstrated that AS-IV can induce the phosphorylation and activation of AMPK in macrophages (Kuang et al., 2025). The mechanisms may involve: 1) Directly activating AMPK by influencing mitochondrial function or increasing the AMP/ATP ratio; 2) Acting through upstream kinases such as LKB1 or CaMKKβ.

##### 4.1.3.2 Role in polarization regulation

The effect of AMPK activation on polarization is context-dependent. In the context of M1 polarization, AMPK activation is generally considered to have anti-inflammatory effects. In the context of M2 polarization, AMPK activation induced by AS-IV may inhibit the M2 phenotype through the following mechanisms: 1) Phosphorylating and inhibiting mTORC1, which echoes the aforementioned PI3K/Akt/mTOR pathway inhibition; 2) Regulating downstream transcription factors such as PGC-1α (peroxisome proliferator-activated receptor γ coactivator 1α), affecting mitochondrial biogenesis and fatty acid oxidation—the latter being the preferred energy source of M2 macrophages; 3) Modulating NADPH oxidase activity, influencing reactive oxygen species (ROS) production, which can act as signaling molecules affecting polarization.

#### 4.1.4 Crosstalk among JAK/STAT3, PI3K/Akt/mTOR, and AMPK pathways

These signaling pathways engage in extensive crosstalk that governs macrophage polarization. The JAK/STAT3 and PI3K/Akt/mTOR axes positively regulate each other through reciprocal activation, while AMPK serves as a negative regulator of both by inhibiting mTORC1, promoting STAT3 degradation, and dampening PI3K/Akt signaling. This crosstalk shapes AS-IV’s effects: coordinated inhibition of STAT3 and PI3K/Akt may synergistically disrupt their mutual reinforcement, and concurrent AMPK activation could further amplify suppression via feed-forward loops. However, potential antagonism must also be considered—sustained AMPK activation may trigger compensatory Akt activation, and prolonged STAT3 inhibition could upregulate other JAK family members. The multi-target nature of AS-IV thus represents both an advantage (coordinated suppression) and a challenge (unpredictable compensatory responses). Elucidating the precise dynamics of this pathway network under AS-IV treatment warrants systems-level investigation using phosphoproteomics and computational modeling.

#### 4.1.5 Other related pathways

AS-IV may also influence other pathways related to macrophage function and polarization, such as:

##### 4.1.5.1 NF-κB pathway

Under pro-inflammatory stimulation, AS-IV typically exhibits an inhibitory effect on NF-κB activation, thereby reducing the production of pro-inflammatory factors (Li et al., 2024). However, in the context of M2 polarization, its effect on NF-κB may be more complex, as certain M2-inducing signals may also involve non-canonical NF-κB pathways.

##### 4.1.5.2 MAPK pathway

Studies suggest that AS-IV may modulate the phosphorylation levels of ERK, p38 MAPK, and JNK, kinases involved in regulating cytokine production and cellular function (Guo C. et al., 2025).

### 4.2 Regulatory role in metabolic reprogramming

Macrophage polarization is inseparable from profound remodeling of metabolic status. By intervening in key metabolic pathways and enzyme activities, AS-IV influences the energy and substrate metabolism of macrophages, thereby altering their functional phenotype.

#### 4.2.1 Regulation of the AMPK/mTOR axis and energy sensing

As previously mentioned, AS-IV activates AMPK and inhibits mTORC1; this regulatory axis represents the core switch of cellular energy metabolism. AMPK activation promotes catabolic pathways (such as fatty acid oxidation—note that the effect of AMPK on glycolysis in macrophages is complex), while mTORC1 inhibition reduces anabolism (such as protein and lipid synthesis). This shift in metabolic state may render macrophages no longer supportive of the sustained tissue repair and immunoregulatory functions required by the M2 phenotype, as the maintenance of M2 function requires active anabolism to produce large quantities of secreted proteins and membrane receptors.

#### 4.2.2 Intervention in mitochondrial function and oxidative phosphorylation

M2 macrophages rely on efficient oxidative phosphorylation (OXPHOS) to generate energy. AS-IV exerts regulatory effects on mitochondrial function and may influence OXPHOS through the following mechanisms: 1) Regulating mitochondrial membrane potential and ROS production; 2) Affecting the activity of electron transport chain complexes; 3) Influencing mitochondrial biogenesis through the AMPK/PGC-1α axis (Zhang S. et al., 2025). Altering the efficiency of OXPHOS may affect the energy supply and functional stability of M2 macrophages.

#### 4.2.3 Inhibition of fatty acid oxidation

Fatty acid oxidation (FAO) is the primary energy source of M2 macrophages, and its enhancement is one of the hallmarks of M2 metabolic reprogramming. The FAO process is transcriptionally regulated by PPARγ and its coactivator peroxisome proliferator-activated receptor γ coactivator 1α (PGC-1α). AS-IV may indirectly downregulate the expression or activity of PPARγ and PGC-1α by inhibiting upstream signals such as STAT6 and PI3K/Akt/mTOR, thereby reducing fatty acid uptake (e.g., downregulating CD36) and the expression of FAO-related enzymes (e.g., CPT1), limiting the energy metabolism of M2 macrophages (Luo et al., 2021).

#### 4.2.4 Effects on amino acid metabolism

Arginine metabolism represents a key metabolic node in macrophage polarization. M1 macrophages highly express iNOS, metabolizing arginine into citrulline and nitric oxide; in contrast, M2 macrophages highly express Arg1, metabolizing arginine into ornithine and polyamines. By downregulating Arg1 expression through the inhibition of such as the STAT6 pathway, AS-IV may alter the metabolic flow of arginine in the TME, reduce the production of immunosuppressive polyamines, and potentially relatively increase arginine available for the iNOS pathway—contributing to the shift toward an M1-like phenotype (Yue et al., 2020).

### 4.3 Potential regulation at the epigenetic level

Epigenetic modifications provide plasticity and long-term memory for the functional state of macrophages, raising the intriguing possibility that stabilizing a shift away from the M2 phenotype may require epigenetic reprogramming. It is important to emphasize that no direct experimental evidence (e.g., ChIP-seq, ATAC-seq, or quantitative histone modification assays) currently demonstrates that AS-IV alters the macrophage epigenome. The following subsections therefore present testable hypotheses regarding how AS-IV might indirectly influence the epigenetic landscape of macrophages through metabolic and enzymatic intermediates.

#### 4.3.1 Metabolite availability and epigenetic modifications

Intermediate products of cellular metabolism serve as substrates or cofactors for many epigenetic modification enzymes, forming a well-documented metabolism-epigenetics axis. Based on this axis, we hypothesize that AS-IV-induced metabolic changes could theoretically impact the following:

##### 4.3.1.1 Acetyl-CoA and histone acetylation

Acetyl-CoA serves as a substrate for histone acetyltransferases (HATs). Because AS-IV may influence glycolysis and fatty acid metabolism, it is plausible that intracellular acetyl-CoA levels and distribution could be altered, potentially affecting histone acetylation marks such as H3K9ac and H3K27ac—marks generally associated with gene activation (Zhou et al., 2017). This remains speculative; direct measurement of acetyl-CoA pools and global or locus-specific acetylation in AS-IV-treated macrophages has not been reported.

##### 4.3.1.2 α-Ketoglutarate and succinate

These TCA cycle intermediates function as cofactors or inhibitors of histone and DNA demethylases. If AS-IV significantly shifts the metabolic profile of macrophages, it could theoretically alter the ratio of these metabolites, thereby influencing histone and DNA methylation levels. Again, this is an inference and has not been experimentally verified in the context of AS-IV.

##### 4.3.1.3 S- adenosylmethionine (SAM)

SAM is the primary methyl donor for DNA and histone methylation. AS-IV might affect the methionine cycle or folate metabolism, leading to altered SAM levels and consequent changes in global or locus-specific methylation. This possibility awaits experimental testing.

#### 4.3.2 Potential effects on epigenetic modification enzymes

A more proximal link between AS-IV and the epigenetic machinery may exist through its known modulation of certain signaling pathways that converge on chromatin-modifying enzymes. For example, AS-IV has been shown to activate AMPK (Qin et al., 2023), which can increase NAD^+^ levels and thereby activate SIRT1, an NAD^+^-dependent class III histone deacetylase. SIRT1 deacetylates both histones and non-histone proteins (such as NF-κB and STAT3), participating in the regulation of inflammation and metabolism. Therefore, we hypothesize that AS-IV-mediated AMPK activation could enhance SIRT1 activity, potentially leading to altered acetylation of specific histones or transcription factors. However, whether AS-IV actually changes SIRT1 activity or histone acetylation in macrophages has not yet been demonstrated. Additionally, AS-IV-mediated inhibition of the JAK/STAT pathway could theoretically reduce the recruitment of downstream epigenetic co-activators, indirectly affecting chromatin states. These mechanistic links remain to be experimentally tested.

#### 4.3.3 Future directions for experimental validation

To move from inference to evidence, future studies should combine genome-wide and locus-specific epigenetic profiling, including ChIP-seq for activating (H3K4me3, H3K27ac) and repressive (H3K27me3) histone marks and ATAC-seq in AS-IV-treated versus vehicle-treated macrophages under M2-polarizing conditions. Candidate M2- and M1-associated loci identified by transcriptomics (e.g., Arg1, Mrc1, Il10, Tnf, Il12b) could then be validated by ChIP-qPCR and bisulfite sequencing for promoter/enhancer histone modifications and DNA methylation changes. To test the proposed metabolism-epigenetics coupling, pharmacological or genetic perturbation of key metabolic nodes—such as glycolysis (2-DG), fatty acid oxidation (etomoxir), or SIRT1 (EX-527)—could be used to determine whether these interventions abrogate AS-IV-induced transcriptional and phenotypic shifts. Finally, adoptive transfer or serial re-stimulation experiments would help assess the stability of the phenotypic shift in the absence of continued treatment, thereby providing evidence for underlying epigenetic memory.

### 4.4 Changes at the cellular functional level

The aforementioned molecular, metabolic, and epigenetic changes ultimately converge into significant alterations in macrophage phenotype and function.

#### 4.4.1 Distinction between inhibition of M2 polarization and true M1 induction

Most current data demonstrate that AS-IV reduces M2 marker expression (CD206, CD163, Arg1) under M2-inducing conditions and partially increases selected M1-like markers *in vitro* (Zhou et al., 2024; Hu et al., 2025). However, these findings largely reflect the inhibition of *de novo* M2 polarization—that is, the prevention of macrophages from acquiring or maintaining an M2 phenotype under M2-polarizing stimuli—rather than the active conversion of pre-existing, fully differentiated M2-like TAMs into M1-like anti-tumor macrophages (Murray et al., 2014). Whether established tumor-resident M2 TAMs are actively converted into M1-like macrophages *in vivo* remains unproven (Mantovani et al., 2017). AS-IV may primarily inhibit the maintenance of the M2 state rather than directly induce M1 polarization; the observed M1-like changes may be secondary to reduced M2-associated signaling or alterations in the tumor microenvironment. This distinction should be kept in mind when interpreting the functional changes described below (Zhang et al., 2026).

#### 4.4.2 Downregulation of M2 marker expression

Macrophages treated with AS-IV exhibit significantly reduced expression of surface markers CD206 (mannose receptor) and CD163 (hemoglobin scavenger receptor) under M2 polarization stimulation, and intracellular arginase-1 activity and protein levels are also markedly decreased (Zhou et al., 2024).

#### 4.4.3 Shift of secretory profile toward pro-inflammatory/anti-tumor direction

AS-IV treatment reduces the ability of macrophages to secrete M2-associated immunosuppressive/chemotactic factors such as IL-10, TGF-β, CCL17, and CCL22. Concurrently, under certain experimental conditions, it may promote the production of M1-associated pro-inflammatory factors such as IL-12 and TNF-α. However, whether this represents true M1 polarization or merely a partial acquisition of selected M1-like features remains unclear and may depend on specific stimulation contexts, dosages, and experimental models (Hu et al., 2025).

#### 4.4.4 Changes in phagocytic function and antigen presentation capacity

The phagocytic function of M2-type macrophages is generally considered to focus on clearing apoptotic cells and debris, with relatively weak antigen presentation capacity. The phenotypic shift induced by AS-IV may enhance the phagocytic ability of macrophages against tumor cells or apoptotic tumor cells and may upregulate the expression of MHC-II molecules and co-stimulatory molecules (such as CD80 and CD86), thereby improving their efficiency in presenting tumor antigens to T cells and promoting the initiation of anti-tumor immune responses (Wang et al., 2026).

#### 4.4.5 Altered ability to directly regulate tumor cell behavior

The conditioned medium of macrophages with an M1-like phenotype demonstrates reduced, or even potentially inhibitory, effects on tumor cell proliferation, invasion, and migration. This is related to the decreased growth-promoting factors and increased pro-inflammatory factors in their secretory profile (Ruan et al., 2026).

In summary, AS-IV forms a multi-level regulatory network by intervening in core signaling pathways such as JAK/STAT, PI3K/Akt/mTOR, and AMPK, influencing macrophage metabolic programs, and potentially regulating epigenetic states—ultimately modulating macrophage polarization by suppressing M2-associated functions and partially promoting M1-like features with anti-tumor potential. This functional modulation serves as the cornerstone for the subsequent systematic remodeling of the tumor immune microenvironment.

## 5 Systemic remodeling of the immune microenvironment mediated by AS-IV

The regulation of TAM polarization by AS-IV is not an isolated event but rather the initiating point that triggers systemic, cascading remodeling of the tumor immune microenvironment. This remodeling encompasses functional and interactional changes in virtually all immune cells and stromal components within the microenvironment (Table 2) (Figure 3).

**TABLE 2:** Systemic remodeling of the hepatocellular carcinoma immune microenvironment by Astragaloside IV.

Microenvironment component	Effects of AS-IV treatment	Underlying mechanisms	Therapeutic implications	References
Adaptive immune system	CD8^+^ cytotoxic T cells	Increased infiltration density; Restored proliferative capacity; Enhanced production of IFN-γ, granzyme B and perforin	Reduced PD-L1 expression on TAMs; Alleviated arginine depletion; Improved cytokine environment	(Xu et al., 2024; Zeng et al., 2026)
	CD4^+^ helper T cells	Shifted balance toward Th1 phenotype; Inhibited Th2 differentiation; Reduced Treg infiltration and function	Decreased IL-4/IL-13 and TGF-β production by TAMs; Reduced CCL22-mediated Treg recruitment	(Gao et al., 2025b; Li et al., 2016)
Innate immune system	Natural killer (NK) cells	Restored cytotoxicity; Upregulated expression of activating receptors (NKG2D)	Reduced TGF-β secretion; Increased IL-12/IL-15 production by M1-like macrophages	(Li et al., 2022a)
	Myeloid-derived suppressor cells (MDSCs)	Decreased recruitment and expansion; Attenuated immunosuppressive function	Inhibited STAT3 signaling; Reduced secretion of CCL2 and CSF-1 by TAMs	(Guo et al., 2025a)
Tumor vasculature	Abnormal angiogenesis	Inhibited endothelial cell proliferation; Reduced vascular density	Decreased production of VEGF, FGF-2 and PDGF by M2-type TAMs	(Hua et al., 2025)
	Vascular normalization	Increased pericyte coverage; Reduced vascular permeability; Improved tumor perfusion	Alleviated hypoxia; Facilitated immune cell infiltration and drug delivery	(Zou et al., 2022)
Extracellular matrix	Fibrotic barrier	Reduced extracellular matrix deposition; Decreased matrix density	Inhibited TGF-β-mediated hepatic stellate cell activation; Altered MMP/TIMP balance	(Zhang et al., 2025b; Zhang et al., 2023)
Tumor cells	Cancer stem cells (CSCs)	Disrupted CSC niches; Reduced stemness properties; Increased differentiation	Decreased secretion of IL-6, PGE2 and nitric oxide by TAMs	(Huang et al., 2023)
	Therapeutic sensitivity	Enhanced response to chemotherapy, radiotherapy and immunotherapy	Improved drug delivery; Restored immune surveillance function	(Ma et al., 2023)

**FIGURE 3:**
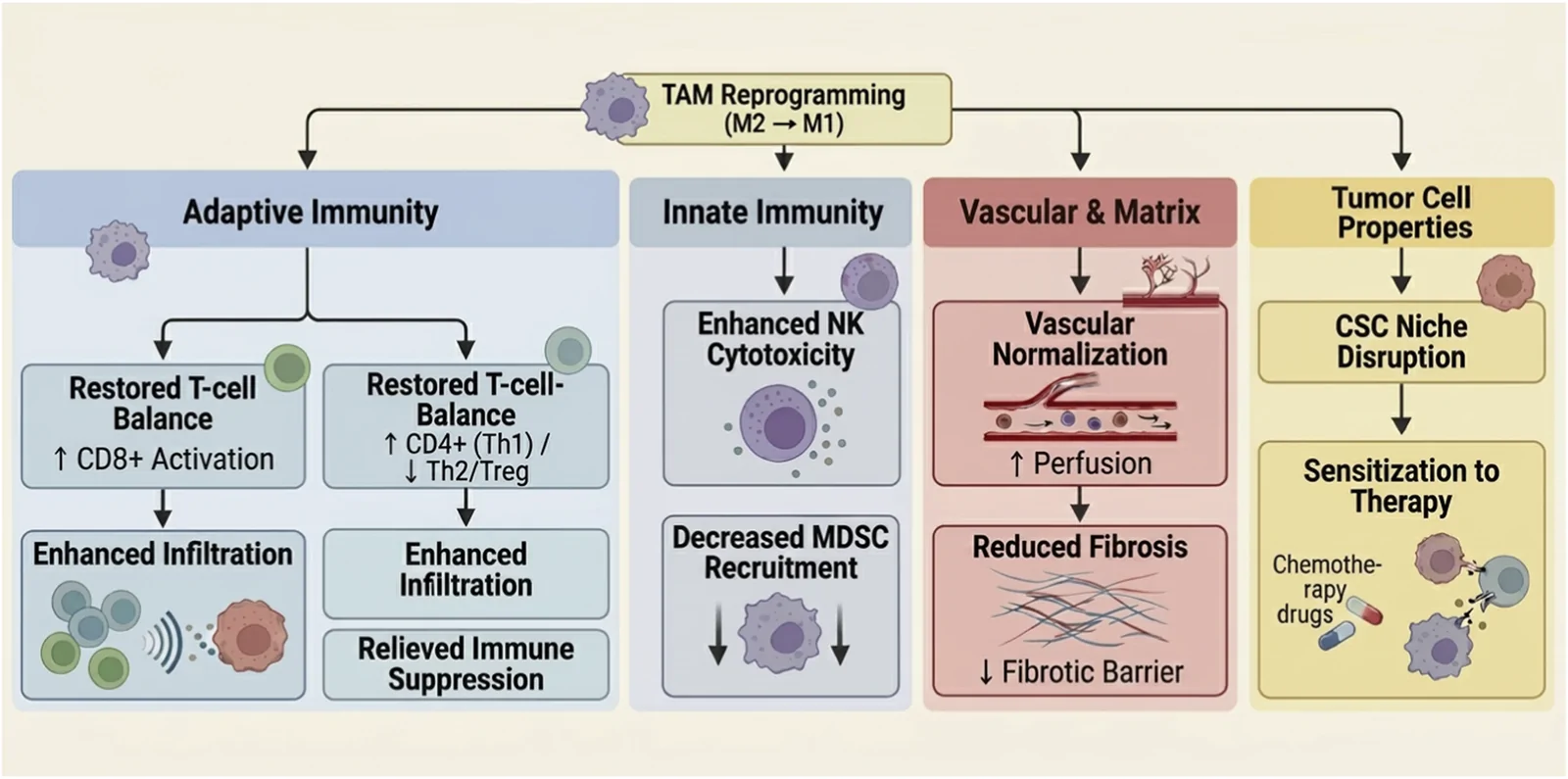
Systemic remodeling of the hepatocellular carcinoma immune microenvironment by astragaloside IV and therapeutic implications. Astragaloside IV targets M2-type TAMs as a critical fulcrum to systematically remodel the HCC immune microenvironment. By reprogramming TAMs toward an M1-like phenotype, AS-IV restores effector T cell function, reduces immunosuppressive cell infiltration, promotes vascular normalization, and ameliorates the fibrotic barrier. This converts “cold” tumors into “hot” tumors, significantly enhancing the efficacy of immune checkpoint inhibitors and other immunotherapies. The combination of AS-IV with immune checkpoint inhibitors represents a promising therapeutic strategy for HCC.

### 5.1 Restoration and activation of the adaptive immune system

#### 5.1.1 Restoration of CD8^+^ cytotoxic T cell function

CD8^+^ T cells represent the primary effector cells of anti-tumor immunity, but they frequently exist in a state of functional exhaustion or anergy within the HCC immunosuppressive microenvironment. Through TAM reprogramming, AS-IV alleviates CD8^+^ T cell suppression via multiple mechanisms:

##### 5.1.1.1 Improving the cytokine environment

Reducing the production of inhibitory cytokines such as IL-10 and TGF-β while potentially augmenting cytokines that promote T cell activation and Th1 differentiation, such as IL-12, thereby creating favorable conditions for the survival and functional execution of CD8^+^ T cells (Xu et al., 2024).

##### 5.1.1.2 Alleviating immune checkpoint-mediated exhaustion

M2-type TAMs represent a major source of PD-L1 within the TME. AS-IV downregulates PD-L1 expression on TAMs, reducing the opportunity for sustained PD-1 activation on T cell surfaces, thereby helping to reverse T cell exhaustion and restore their proliferative capacity and ability to produce effector molecules (such as IFN-γ, granzyme B, and perforin) (Zeng et al., 2026).

##### 5.1.1.3 Relieving metabolic suppression

By downregulating Arg1 expression in TAMs, AS-IV alleviates arginine depletion within the TME, thereby helping to restore the metabolic adaptability and function of T cells.

##### 5.1.1.4 Promoting T cell infiltration and survival

The altered macrophage secretory profile may affect the chemokine network—for example, reducing chemokines that recruit Tregs such as CCL22 while potentially augmenting chemokines that recruit effector T cells such as CXCL9, CXCL10, and CXCL11—thereby altering the composition and spatial distribution of T cells within the tumor (Yang et al., 2024). Animal experiments have demonstrated that following AS-IV treatment, the infiltration density of CD8^+^ T cells in tumor tissue significantly increases, and their effector function is enhanced.

#### 5.1.2 Remodeling of CD4^+^ helper T cell subset balance

CD4^+^ T cells play a critical role in orchestrating anti-tumor immune responses. The microenvironmental changes induced by AS-IV favor anti-tumor Th1 responses while simultaneously suppressing immunosuppressive Th2 and Treg responses.

##### 5.1.2.1 Promoting Th1 differentiation

The relative increase in IL-12 levels and the attenuated inhibitory environment are conducive to the differentiation of naïve CD4^+^ T cells into IFN-γ-secreting Th1 cells under stimulation by antigen-presenting cells such as DCs, thereby enhancing cellular immune responses (Gao R. et al., 2025).

##### 5.1.2.2 Inhibiting Th2 differentiation

IL-4 and IL-13 secreted by M2-type TAMs are key drivers of Th2 differentiation. By inhibiting M2 polarization, AS-IV reduces the source of these cytokines, thereby indirectly suppressing Th2 responses—which are generally associated with humoral immunity and certain pro-tumorigenic effects.

##### 5.1.2.3 Limiting tregs proliferation and function

The induction, expansion, and functional maintenance of Tregs are highly dependent on TGF-β and IL-10. By reducing the production of these factors by TAMs, AS-IV directly attenuates the survival environment of Tregs (Li et al., 2016). Concurrently, altered macrophages may reduce CCL22 secretion, thereby decreasing chemotactic recruitment of Tregs. The relative reduction in Treg numbers and diminished function further alleviate the suppression of effector T cells.

### 5.2 Activation of the innate immune system

#### 5.2.1 Restoration of natural killer cell function

NK cells represent the first line of anti-tumor defense in the innate immune system, but their function is similarly suppressed in the HCC microenvironment.

##### 5.2.1.1 Relieving inhibitory signals

TGF-β secreted by M2-type TAMs is one of the main inhibitory factors for NK cell function; it can downregulate the expression of NK cell activating receptors (such as NKG2D) and inhibit their cytotoxicity and cytokine production. By reducing TGF-β production, AS-IV helps restore the killing potential of NK cells (Li S. et al., 2022).

##### 5.2.1.2 Providing activation signals

Cytokines such as IL-12, IL-15, and IL-18 produced by M1-type macrophages can effectively activate and maintain NK cell function. The macrophage phenotypic transition induced by AS-IV may increase the production of these cytokines, providing positive stimulation for NK cells.

#### 5.2.2 Effects on MDSCs

MDSCs represent another heterogeneous cell population with potent immunosuppressive capacity. Close interactions and positive feedback loops exist between TAMs and MDSCs.

##### 5.2.2.1 Interfering with recruitment and expansion

TAMs can secrete factors such as CCL2 and CSF-1 to recruit MDSC precursors and promote their expansion. By inhibiting M2-type TAMs, AS-IV may reduce the secretion of these factors, thereby limiting the accumulation of MDSCs at tumor sites (Guo L. et al., 2025).

##### 5.2.2.2 Weakening immunosuppressive function

The suppressive function of MDSCs partially depends on the activation of such as the STAT3 pathway. The inhibitory effects of AS-IV on these pathways may similarly extend to MDSCs, reducing their production of Arg1, iNOS, and ROS, and weakening their ability to suppress T cells and NK cells.

### 5.3 Normalization of the tumor vascular system and extracellular matrix

#### 5.3.1 Inhibition of abnormal angiogenesis

HCC is a highly vascularized tumor, and abnormal neovascularization not only provides nutrients for tumor growth but also constitutes physical and functional barriers to immune cell infiltration.

##### 5.3.1.1 Reducing pro-angiogenic factors

M2-type TAMs are a major source of pro-angiogenic factors such as VEGF, FGF-2, and PDGF. By inhibiting M2 polarization, AS-IV directly cuts off an important supply source of these factors, thereby inhibiting abnormal endothelial cell proliferation and angiogenesis (Hua et al., 2025).

##### 5.3.1.2 Promoting vascular normalization

Studies suggest that certain immunomodulators, while inhibiting excessive angiogenesis, may induce structural and functional “normalization” of remaining blood vessels—manifested as increased pericyte coverage, reduced vascular permeability, and improved perfusion. Although direct evidence for AS-IV in this regard still needs to be accumulated, theoretically, by regulating TAMs and improving hypoxia, it may contribute to the formation of vascular networks more conducive to immune cell migration and drug delivery (Zou et al., 2022).

#### 5.3.2 Amelioration of the fibrotic barrier

HCC often occurs against a background of chronic liver disease (such as cirrhosis), accompanied by significant fibrosis and extracellular matrix remodeling, forming physical barriers and generating immunosuppressive signals.

##### 5.3.2.1 Regulating the macrophage-hepatic stellate cell axis

Activated hepatic stellate cells (HSCs) are the main cells producing extracellular matrix. M2-type TAMs activate and maintain the activated state of HSCs by secreting factors such as TGF-β and PDGF. Conversely, activated HSCs also secrete factors to recruit and polarize macrophages. By inhibiting M2-type TAMs, AS-IV may break this positive feedback loop, reducing activation signals to HSCs (Zhang L. et al., 2025).

##### 5.3.2.2 Reducing matrix deposition

TGF-β is the most potent pro-fibrotic factor. By reducing TAM-derived TGF-β, AS-IV may directly attenuate the fibrotic process. Additionally, M2-type TAMs themselves secrete certain matrix remodeling enzymes. Phenotypically transformed macrophages may alter the balance between MMPs and their inhibitors, affecting matrix degradation and deposition, thereby reducing matrix density and facilitating immune cell infiltration (Zhang et al., 2023).

### 5.4 Indirect alterations of tumor cell properties

#### 5.4.1 Disruption of CSCs niches

CSCs are resistant to conventional therapies and are considered the root of recurrence and metastasis. M2-type TAMs provide microenvironments that maintain stemness and self-renewal for CSCs by secreting factors such as IL-6, PGE2, and nitric oxide.

##### 5.4.1.1 Cutting off support signals

After AS-IV inhibits M2-type TAMs, the secretion of these supporting factors is reduced, potentially causing CSCs to lose niche support and be forced to transition to a more differentiated, treatment-sensitive cell state (Huang et al., 2023).

##### 5.4.1.2 Enhancing immune system recognition of CSCs

M1-like macrophages may enhance phagocytosis and antigen presentation of CSCs, thereby stimulating specific immune responses against CSCs.

#### 5.4.2 Sensitization to conventional therapies

The systemic improvement of the TME by AS-IV may enhance the efficacy of chemotherapy, targeted therapy, and even radiotherapy.

##### 5.4.2.1 Improving drug delivery

Vascular normalization and matrix remodeling may increase the penetration and distribution of chemotherapeutic/targeted drugs within tumor tissue (He et al., 2025).

##### 5.4.2.2 Reversing immunosuppression-induced therapeutic resistance

The efficacy of many conventional treatments partially depends on the participation of the immune system (e.g., immunogenic cell death). The restored immune surveillance function mediated by AS-IV may enhance the anti-tumor immune responses induced by these treatments, generating abscopal effects and immune memory (Ma et al., 2023).

##### 5.4.2.3 Synergistic induction of cell death

AS-IV itself has the effects of inducing tumor cell apoptosis and endoplasmic reticulum stress, potentially producing synergistic effects with certain chemotherapeutic drugs.

In summary, AS-IV leverages TAMs as a critical fulcrum to pry the entire HCC immune microenvironment from an “immunosuppressive” state toward a “permissive” or even “activated” state. This systemic remodeling not only restores anti-tumor immune responses but also improves the physical and metabolic microenvironment of the tumor, creating favorable conditions for the combined application of multiple therapeutic strategies.

## 6 Translational potential and challenges of AS-IV in HCC therapy

### 6.1 Synergistic potential as an immunotherapy sensitizer

ICIs, represented by PD-1/PD-L1 inhibitors, have revolutionized the treatment landscape of HCC, but the objective response rate remains limited (approximately 15%–20%), and primary or acquired resistance persists (Li Q. et al., 2022). The tumor immunosuppressive microenvironment, particularly the enrichment of M2-type TAMs, represents a critical contributor to the “cold tumor” phenotype and ICI resistance. Therefore, the combination of AS-IV with ICIs has a strong theoretical synergistic basis (Table 3).

**TABLE 3:** Translational strategies and challenges of Astragaloside IV in hepatocellular carcinoma therapy.

Therapeutic strategy	Rationale	Advantages	Current challenges	Potential solutions	References
Combination with immune checkpoint inhibitors (ICIs)	Converts “cold tumors” to “hot tumors”; Overcomes multiple immunosuppressive mechanisms; Regulates PD-L1 expression on TAMs	Synergistic anti-tumor effects; Reduced ICI resistance; Improved objective response rates	Optimal dosing schedule; Biomarkers for patient selection	Phase II/III clinical trials; Companion diagnostic development	(Li et al., 2022b; Liu et al., 2024)
Combination with other immunotherapies	Adoptive cell therapies (CAR-T, TIL): Improved survival and infiltration of infused T cells; Cancer vaccines: Enhanced DC function and antigen presentation	Expands the scope of immunotherapy; Addresses limitations of single-agent approaches	Manufacturing complexity; Cost-effectiveness	Off-the-shelf cell products; Neoantigen-based personalized vaccines	(Chohan et al., 2023; Kaczmarek et al., 2023)
Nano-delivery systems	Improves aqueous solubility; Protects from degradation; Achieves passive targeting via EPR effect	Prolonged blood circulation time; Increased tumor accumulation; Reduced systemic toxicity	Scalability; Long-term safety; Regulatory hurdles	GMP-compliant manufacturing; Comprehensive toxicological evaluations	(Kong et al., 2024)
Active targeting strategies	TAM targeting (mannose, galactose); Tumor vasculature targeting (RGD peptides); Tumor cell targeting (folic acid)	Enhanced local drug concentration; Improved reprogramming effects; Reduced off-target effects	Ligand stability; Immunogenicity of targeting moieties	Multivalent ligand design; Biodegradable carrier materials	(Yang et al., 2020)
Stimuli-responsive drug release systems	pH-sensitive; Enzyme-sensitive; ROS-sensitive; Dual/multiple responsiveness	Precise spatiotemporal drug release; Maximized therapeutic efficacy; Minimized side effects	Complexity of design; *In vivo* response variability	Smart nanocarriers with multiple response mechanisms	(Yang et al., 2025b)
Combination drug delivery nano-platforms	Co-delivery of AS-IV with ICIs or small molecule inhibitors	Synchronized synergistic therapy; Simplified dosing regimens; Improved pharmacokinetic profiles	Drug-loading efficiency; Controlled release ratio	Rational drug pairing; Optimized carrier formulation	(Shim et al., 2022)

#### 6.1.1 Mechanisms of synergistic action

##### 6.1.1.1 Converting “cold tumors” to “hot tumors

The premise of ICI action is the presence of a certain number of tumor-infiltrating lymphocytes (TILs) within the tumor, albeit with suppressed function. By reversing TAM polarization, augmenting effector T cell infiltration, and enhancing T cell function, AS-IV can effectively increase immune cell infiltration and inflammatory signals in tumors, providing the necessary immune cell basis and environmental conditions for ICIs to exert their effects (Liu et al., 2024). Preclinical studies have demonstrated that AS-IV pretreatment or combination therapy can significantly increase tumor sensitivity to PD-1 antibodies.

##### 6.1.1.2 Overcoming multiple immunosuppressive mechanisms

ICIs primarily block the PD-1/PD-L1 pathway, but tumors may compensate by upregulating other immune checkpoints (such as cytotoxic T-lymphocyte-associated protein 4 (CTLA-4), lymphocyte-activation gene 3 (LAG-3), and T-cell immunoglobulin and mucin-domain containing-3 (TIM-3)) or augmenting the function of other suppressor cells such as Tregs and MDSCs. The systemic immunomodulatory effects of AS-IV can simultaneously act on these compensatory mechanisms—for example, reducing Treg recruitment and attenuating MDSC function—thereby preventing or delaying the development of resistance (Yang et al., 2024).

##### 6.1.1.3 Regulating PD-L1 expression on TAMs

Intriguingly, while inhibiting M2 polarization, AS-IV may selectively downregulate immunosuppressive PD-L1 expression on M2-type TAMs without affecting or potentially augmenting the expression of necessary co-stimulatory molecules on antigen-presenting cells, thereby more precisely releasing the immune brake rather than comprehensively inhibiting immune checkpoints (Zeng et al., 2026).

#### 6.1.2 Combination potential with other immunotherapies

In addition to combination with ICIs, AS-IV may also synergize with adoptive cell therapies, cancer vaccines, and other immunotherapeutic modalities.

##### 6.1.2.1 Adoptive cell therapies

Such as chimeric antigen receptor T cells (CAR-T) or TIL therapies. The improved TME mediated by AS-IV (reduced inhibitory factors, improved vasculature and stroma) may facilitate the survival, infiltration, and functional persistence of infused T cells at tumor sites (Chohan et al., 2023).

##### 6.1.2.2 Therapeutic vaccines

AS-IV enhances DC function and antigen presentation, potentially augmenting the efficiency of tumor vaccines in eliciting specific T cell responses (Kaczmarek et al., 2023).

#### 6.1.3 Strategic considerations for combining AS-IV with first-line HCC agents

Despite strong theoretical synergies, the translation of AS-IV-based combinations requires careful consideration of timing, dose ratios, and potential pharmacological antagonism, particularly given AS-IV’s modulation of the PI3K/Akt/mTOR pathway, which overlaps with the mechanisms of several first-line HCC drugs (Table 4).

**TABLE 4:** Potential synergistic and antagonistic relationships of AS-IV in combination therapy.

Combination partner	Proposed synergistic mechanism	Potential antagonism/overlap risk	Suggested strategy/open questions
Anti-PD-1/PD-L1	AS-IV increases TILs, reduces M2-TAMs, converts cold to hot tumors (Liu et al., 2024); may downregulate PD-L1 on M2-TAMs selectively (Zeng et al., 2026)	Possible reduction in PD-L1 targets for anti-PD-L1 antibodies; increased irAEs (Li et al., 2022b)	Explore sequential (AS-IV lead-in → ICI) vs. concurrent regimens; monitor irAEs; define optimal AS-IV dose for immunomodulation
Lenvatinib	Dual inhibition of angiogenesis and PI3K/Akt/mTOR; AS-IV counteracts hypoxia-induced M2 polarization (Yang et al., 2024)	Overlapping PI3K/Akt/mTOR suppression may increase toxicity or impair anti-tumor immunity; VEGF inhibition may reduce drug delivery	Preclinical testing of combination index; consider staggered dosing; evaluate effects on immune subsets and tumor oxygenation
Sorafenib	Similar to lenvatinib; AS-IV may alleviate sorafenib-induced immunosuppression (Yang et al., 2024)	Overlapping pathway inhibition; sorafenib can decrease M1 macrophage viability; additive hepatotoxicity risk	*In vivo* testing in fibrotic HCC models; careful liver function monitoring; potential for AS-IV to rescue sorafenib-related immune dysfunction
CAR-T/Adoptive Cell Therapy	AS-IV remodels TME, reduces inhibitory cytokines, improves T cell infiltration and persistence (Chohan et al., 2023)	Over-stimulation may increase CRS risk; AS-IV effects on B cell/antibody responses may impact lymphodepletion regimens	Determine optimal timing relative to T cell infusion; evaluate effects on expansion and persistence of CAR-T cells

##### 6.1.3.1 Anti-PD-1/PD-L1 therapy

###### 6.1.3.1.1 Optimal timing and sequence

Preclinical evidence indicates that AS-IV pretreatment can convert an immune “cold” tumor into a “hot” one, suggesting that a lead-in period of AS-IV monotherapy followed by ICI administration may maximize synergism (Liu et al., 2024). Concurrent administration, while potentially more convenient, might prematurely expose reinvigorated T cells to an incompletely remodeled TME, risking suboptimal activation or even T-cell exhaustion. Future clinical trials should explore both sequential (AS-IV → ICI) and concurrent regimens with integrated biomarker analysis (e.g., CD8^+^ T-cell infiltration after lead-in).

###### 6.1.3.1.2 Dose ratio

No optimal dose ratio has been established; however, preclinical models suggest that moderate immunomodulatory doses of AS-IV (capable of M2→M1 polarization without systemic immunosuppression) should be combined with standard ICI doses. Overly high AS-IV doses could theoretically suppress necessary immune checkpoints on effector cells or increase immune-related adverse events (irAEs). Clinical combination studies should include AS-IV dose escalation with pharmacokinetic/pharmacodynamic monitoring.

###### 6.1.3.1.3 Antagonistic risks

AS-IV’s ability to downregulate PD-L1 on M2-TAMs may theoretically reduce the number of PD-L1 targets available for anti-PD-L1 antibodies, potentially diminishing drug occupancy and efficacy (Zeng et al., 2026). However, this effect is likely offset by the global increase in CD8^+^ T-cell infiltration and function. A more relevant risk is the overlapping toxicity: AS-IV-enhanced immune activation could increase the incidence or severity of irAEs when combined with ICIs, necessitating careful safety monitoring (Li Q. et al., 2022).

##### 6.1.3.2 Multitargeted tyrosine kinase inhibitors (TKIs)

###### 6.1.3.2.1 Overlap in PI3K/Akt/mTOR inhibition

Lenvatinib and sorafenib inhibit VEGFR and FGFR, both of which signal partly through the PI3K/Akt/mTOR axis (Yang et al., 2024). AS-IV also suppresses this pathway via multiple upstream targets (e.g., PTEN upregulation, PI3K inhibition) (Zeng et al., 2026). Combined inhibition may result in:

1. Potentiated anti-angiogenic and anti-tumor effects, through deeper suppression of the PI3K/Akt/mTOR pathway and more complete blockade of compensatory signaling.
2. Potential antagonism or excessive toxicity: If the pathway is overly suppressed, normal tissue homeostasis (e.g., hepatic regeneration, immune cell signaling) may be compromised, leading to increased adverse effects such as fatigue, hepatotoxicity, or impaired wound healing. Additionally, some TKIs may increase hypoxia in the TME, which could paradoxically promote M2 polarization—an effect that AS-IV may partially counteract through macrophage reprogramming (Yang et al., 2024).

###### 6.1.3.2.2 Strategic framework

Preclinical experiments should systematically evaluate the effect of AS-IV on lenvatinib/sorafenib IC50 values in HCC cell lines under normoxic and hypoxic conditions, as well as the net immune-modulatory outcome in co-culture systems. A staggered schedule (e.g., AS-IV after TKI-induced vascular normalization) may warrant exploration. At present, no clinical evidence supports combination, and the risk-benefit ratio must be clarified before proceeding.

### 6.2 Development strategies for novel delivery systems

The inherent pharmacokinetic limitations of AS-IV (low oral bioavailability, lack of targeting) represent one of the major bottlenecks in its clinical translation. The development of advanced delivery systems is critical to enhancing its efficacy and safety.

#### 6.2.1 Nano-delivery systems

##### 6.2.1.1 Types

Including liposomes, polymeric nanoparticles (PLGA, chitosan), solid lipid nanoparticles, mesoporous silica nanoparticles, nanoemulsions, and others. These systems can effectively improve the water solubility of AS-IV, protect it from degradation, and achieve passive targeting using the enhanced permeability and retention (EPR) effect of solid tumors (Kong et al., 2024).

##### 6.2.1.2 Advantages

Prolonging blood circulation time, increasing drug accumulation at tumor sites, and reducing exposure to normal tissues, thereby potentially improving the therapeutic index.

#### 6.2.2 Active targeting strategies

Modifying the surface of nanocarriers with targeting ligands to achieve cell- or tissue-specific delivery.

##### 6.2.2.1 Targeting TAMs

Modifying with mannose, galactose, or phosphatidylserine analogs to achieve specific drug accumulation within TAMs by leveraging the highly expressed mannose receptors, scavenger receptors, or Tim-4 receptors on TAM surfaces—improving local concentration, enhancing reprogramming effects, and reducing systemic impacts (Yang et al., 2020).

##### 6.2.2.2 Targeting tumor vasculature

Modifying with RGD peptides to target integrin αvβ3, which is highly expressed on tumor neovascular endothelium.

##### 6.2.2.3 Targeting tumor cells

Modifying with folic acid to target tumor cells with high folate receptor expression.

#### 6.2.3 Stimuli-responsive drug release systems

Designing “smart” carriers that respond to the unique characteristics of the TME to achieve precise drug release at target sites.

##### 6.2.3.1 pH-sensitive

The TME and TAM lysosomes are weakly acidic. Using pH-sensitive chemical bonds (such as hydrazone bonds) to link drugs, or selecting pH-sensitive carrier materials (such as PEGylated liposomes), for rapid drug release in acidic environments (Gu et al., 2024).

##### 6.2.3.2 Enzyme-sensitive

Certain enzymes (such as MMPs and cathepsins) have excessively high activity at tumor sites or within TAMs. Designing peptide segments specifically cleaved by these enzymes to link drugs or serve as part of nanocarriers.

##### 6.2.3.3 Reactive oxygen species (ROS)-sensitive

ROS levels are elevated in the TME and within activated immune cells. Using materials containing ROS-sensitive bonds such as thioketals.

##### 6.2.3.4 Dual/multiple responsiveness

Combining two or more response mechanisms for more precise control.

#### 6.2.4 Combination drug delivery nano-platforms

Constructing nano-platforms for co-delivery of AS-IV with ICIs (such as anti-PD-1 antibodies) or other small molecule inhibitors (such as STAT3 inhibitors) to achieve spatiotemporally synchronized synergistic therapy, simplifying dosing regimens, and potentially altering drug distribution and pharmacokinetics through carrier properties (Shim et al., 2022).

#### 6.2.5 Challenges in fibrotic/cirrhotic liver: penetration, long-term safety, and immunogenicity

The clinical application of nano-delivery systems for AS-IV in HCC must also address the pathology of fibrotic/cirrhotic livers. First, dense extracellular matrix and elevated interstitial pressure severely limit nanoparticle penetration, causing perivascular trapping that compromises drug delivery to deeper tumor regions. Second, the long-term fate and toxicity of carrier materials remain poorly characterized; PLGA degradation can release acidic byproducts that exacerbate local inflammation, while inorganic nanoparticles may accumulate in Kupffer cells and hepatocytes, disrupting metabolic and immune functions. Third, repeated administration of targeted nanocarriers carries immunogenicity risks, including anti-polymer antibody production, complement activation-related pseudoallergy, and accelerated blood clearance—effects likely amplified in patients with chronic liver inflammation. Addressing these challenges requires optimizing nanocarrier design for enhanced stromal penetration, evaluating biocompatibility in fibrotic liver models, and exploring biomimetic strategies that minimize off-target recognition while preserving TAM-specific accumulation.

### 6.3 Safety and toxicity

The clinical translation of AS-IV for HCC requires thorough safety evaluation. Preclinical studies show a favorable safety profile, with an LD_50_ exceeding 80 mg/kg (i.v.) in rodents, suggesting a wide therapeutic window (Chen et al., 2025). However, several concerns merit attention, particularly given that most HCC patients have underlying cirrhosis and compromised hepatic function.

First, high-dose AS-IV has been associated with mild, reversible hepatotoxicity in some animal models, manifesting as transient ALT/AST elevations and subtle histopathological changes—raising concerns for patients with pre-existing hepatic impairment.

Second, AS-IV’s immunomodulatory effects carry theoretical risks of immune-related adverse events, especially when combined with immune checkpoint inhibitors. Excessive M1-like macrophage activation could potentially trigger cytokine release syndrome or exacerbate autoimmunity in susceptible individuals.

Third, long-term safety in cirrhosis remains poorly characterized. Cirrhotic livers exhibit altered drug-metabolizing enzyme activity, compromised biliary excretion, and disrupted sinusoidal architecture, all of which may alter AS-IV pharmacokinetics and toxicity. Dedicated studies in cirrhotic models and patients with Child-Pugh A/B cirrhosis are warranted.

Finally, potential drug-drug interactions must be assessed, given AS-IV’s modulation of pathways (e.g., PI3K/Akt, AMPK) that intersect with those targeted by concurrent therapies (e.g., sorafenib, lenvatinib, ICIs). Comprehensive toxicological assessments in relevant disease models are essential before clinical evaluation.

## 7 Conclusion and prospects

A central feature of the HCC immunosuppressive microenvironment resides in the predominant role of M2-type TAMs, and targeting their phenotypic modulation has emerged as a pivotal therapeutic strategy. This review systematically summarizes current evidence indicating that AS-IV, as a multi-target immunomodulatory agent, can modulate this process through multi-level mechanisms. These concerted effects modulate macrophage polarization by suppressing M2-associated functions and partially promoting M1-like features with anti-tumor potential, which in turn contribute to the remodeling of the immune microenvironment. This multi-level regulatory network exemplifies the scientific rationale underlying the traditional Chinese medicine concept of “holistic modulation.” Looking ahead, the development of Astragaloside IV as an immunotherapeutic sensitizer, particularly when combined with ICIs, holds substantial translational potential. However, several challenges must be addressed, including overcoming pharmacokinetic limitations, developing targeted delivery systems, and undergoing rigorous clinical validation.

## References

[B1] Avila M. A. Berasain C. (2019). Targeting CCL2/CCR2 in tumor-infiltrating macrophages: a tool emerging out of the box against hepatocellular carcinoma. Cell Mol. Gastroenterol. Hepatol. 7 (2), 293–294. 10.1016/j.jcmgh.2018.11.002 30529279 PMC6354282

[B2] Baglieri J. Brenner D. A. Kisseleva T. (2019). The role of fibrosis and liver-associated fibroblasts in the pathogenesis of hepatocellular CarcinomaInternational. Journal Molecular Sciences 20 (7), E1723. 10.3390/ijms20071723 30959975 PMC6479943

[B3] Butt M. A. Aby E. S. Debes J. D. (2026). The global epidemiology of hepatocellular carcinoma. Hepatol. Commun. 10 (5), e0932. 10.1097/HC9.0000000000000932 41974030 PMC13075906

[B4] Cao H. Wang S. (2024). G-CSF promotes the development of hepatocellular carcinoma by activating the PI3K/AKT/mTOR pathway in TAM. Aging (Albany NY) 16 (13), 10799–10812. 10.18632/aging.205922 38967628 PMC11272108

[B5] Chen X. Yang M. Yin J. Li P. Zeng S. Zheng G. (2022). Tumor-associated macrophages promote epithelial-mesenchymal transition and the cancer stem cell properties in triple-negative breast cancer through CCL2/AKT/β-catenin signaling. Cell Commun. Signal 20 (1), 92. 10.1186/s12964-022-00888-2 35715860 PMC9205034

[B6] Chen C. Bu X. Deng L. Xia J. Wang X. Chen L. (2025). Astragaloside IV as a promising therapeutic agent for liver diseases: current landscape and future perspectives. Front. Pharmacol. 16, 1574154. 10.3389/fphar.2025.1574154 40337517 PMC12055773

[B7] Cheng K. Cai N. Zhu J. Yang X. Liang H. Zhang W. (2022). Tumor-associated macrophages in liver cancer: from mechanisms to therapy. Cancer Commun. (Lond) 42 (11), 1112–1140. 10.1002/cac2.12345 36069342 PMC9648394

[B8] Chohan K. L. Siegler E. L. Kenderian S. S. (2023). CAR-T cell therapy: the efficacy and toxicity balance. Curr. Hematol. Malig. Rep. 18 (2), 9–18. 10.1007/s11899-023-00687-7 36763238 PMC10505056

[B9] Cui Y. Chen J. Zhang Z. Shi H. Sun W. Yi Q. (2023). The role of AMPK in macrophage metabolism, function and polarisation. J. Transl. Med. 21 (1), 892. 10.1186/s12967-023-04772-6 38066566 PMC10709986

[B10] Daskalaki M. G. Lapi I. Hurst A. E. Al-Qahtani A. Vergadi E. Tsatsanis C. (2025). Epigenetic and metabolic regulation of macrophage responsiveness and memory. J. Immunol. 214 (11), 2812–2821. 10.1093/jimmun/vkaf135 40591855

[B11] Dopazo C. Søreide K. Rangelova E. Mieog S. Carrion-Alvarez L. Diaz-Nieto R. (2024). Hepatocellular carcinoma. Eur. J. Surg. Oncol. 50 (1), 107313. 10.1016/j.ejso.2023.107313 38086315

[B12] Fiorucci S. Distrutti E. (2015). Bile acid-activated receptors, intestinal microbiota, and the treatment of metabolic DisordersTrends in. Molecular Medicine 21 (11), 702–714. 10.1016/j.molmed.2015.09.001 26481828

[B13] Fortelny N. Farlik M. Fife V. Gorki A. D. Lassnig C. Maurer B. (2024). JAK-STAT signaling maintains homeostasis in T cells and macrophages. Nat. Immunol. 25 (5), 847–859. 10.1038/s41590-024-01804-1 38658806 PMC11065702

[B14] Gao X. Hao W. Wang Y. Wu X. Zhu F. Zhang Y. (2025a). Molecular mechanisms of astragaloside-IV in hepatocellular carcinoma therapy: a systematic review. BMC Cancer 25 (1), 1407. 10.1186/s12885-025-14758-w PMC12400760 40890667

[B15] Gao R. Wu Z. Dang W. Yang T. Chen J. Cheng H. (2025b). Th1/Th2 immune imbalance in the spleen of mice induced by hypobaric hypoxia stimulation and therapeutic intervention of astragaloside IV. Int. J. Mol. Sci. 26 (6), 2584. 10.3390/ijms26062584 PMC11942621 40141225

[B16] Gu X. Zhu Y. Su J. Wang S. Su X. Ding X. (2024). Lactate-induced activation of tumor-associated fibroblasts and IL-8-mediated macrophage recruitment promote lung cancer progression. Redox Biol. 74, 103209. 10.1016/j.redox.2024.103209 38861833 PMC11215341

[B17] Guo H. Zhao J. Wu C. (2022). Astragaloside IV-enhanced Anti-hepatocarcinoma immunity of dendritic cells. Asian J. Surg. 45 (5), 1216–1218. 10.1016/j.asjsur.2022.01.074 35241335

[B18] Guo L. Yi J. Zhang A. Zheng X. Wang M. Yang F. (2025a). Zhenqi Fuzheng Granule targets the SCFAs-GPR109A axis to enhance PD-1 antibody efficacy *via* immunometabolic remodeling in colorectal cancer. Phytomedicine 148, 157312. 10.1016/j.phymed.2025.157312 41038145

[B19] Guo C. Li Y. Yang R. Xie M. Chen X. Che Z. (2025b). Astragaloside IV attenuates glucocorticoid-induced osteoclastogenesis and bone loss *via* the MAPK/NF-κB pathway. BMC Complement. Med. Ther. 25 (1), 48. 10.1186/s12906-025-04793-2 PMC11818135 39934767

[B20] He Q. Xiang L. Luo Y. Wang R. Zheng C. Gao Y. (2025). Tumor-associated macrophages in colon cancer immunotherapy: mechanisms, natural product interventions, and microenvironment remodeling. Front. Immunol. 16, 1642091. 10.3389/fimmu.2025.1642091 40873588 PMC12378638

[B21] Hou S. Zhao Y. Chen J. Lin Y. Qi X. (2024). Tumor-associated macrophages in colorectal cancer metastasis: molecular insights and translational perspectives. J. Transl. Med. 22 (1), 62. 10.1186/s12967-024-04856-x 38229160 PMC10792812

[B22] Hu Z. Sui Q. Jin X. Shan G. Huang Y. Yi Y. (2024). IL6-STAT3-C/EBPβ-IL6 positive feedback loop in tumor-associated macrophages promotes the EMT and metastasis of lung adenocarcinoma. J. Exp. Clin. Cancer Res. 43 (1), 63. 10.1186/s13046-024-02989-x PMC10903044 38424624

[B23] Hu Y. Kuang M. Song H. Tan Y. Zhou F. Pei G. (2025). Astragaloside IV prevents liver fibrosis by blocking glycolysis-mediated macrophage M1 polarization. Eur. J. Pharmacol. 995, 177353. 10.1016/j.ejphar.2025.177353 39971227

[B24] Hua S. Zhang H. Li J. Zhou X. Zhang S. Zhu Y. (2025). Astragaloside IV ameliorates atherosclerosis by targeting TAK1 to suppress endothelial cell proinflammatory activation. Int. Immunopharmacol. 146, 113842. 10.1016/j.intimp.2024.113842 39706043

[B25] Huang P. Li H. Ren L. Xie H. Chen L. Liang Y. (2023). Astragaloside IV enhances the sensitivity of breast cancer stem cells to paclitaxel by inhibiting stemness. Transl. Cancer Res. 12 (12), 3703–3717. 10.21037/tcr-23-1885 38193000 PMC10774038

[B26] Kaczmarek M. Poznańska J. Fechner F. Michalska N. Paszkowska S. Napierała A. (2023). Cancer vaccine therapeutics: limitations and Effectiveness-A literature review. Cells 12 (17), 2159. 10.3390/cells12172159 37681891 PMC10486481

[B27] Kong C. Sun J. Hu X. Li G. Wu S. (2024). A tumor targeted nano micelle carrying astragaloside IV for combination treatment of bladder cancer. Sci. Rep. 14 (1), 17704. 10.1038/s41598-024-66010-3 39085255 PMC11291986

[B28] Kuang G. Zhao Y. Wang L. Wen T. Liu P. Ma B. (2025). Astragaloside IV alleviates acute hepatic injury by regulating macrophage polarization and pyroptosis *via* activation of the AMPK/SIRT1 signaling pathway. Phytother. Res. 39 (2), 733–746. 10.1002/ptr.8403 39660635

[B29] Kumar M. Singh S. B. Vasyliv I. Habte F. Kalita M. Alam I. S. (2025). B7-H4 ImmunoPET imaging tracks tumor-associated macrophage changes in prostate cancer. Mol. Pharm. 22 (12), 7400–7411. 10.1021/acs.molpharmaceut.5c00637 41122911

[B30] Kzhyshkowska J. Shen J. Larionova I. (2024). Targeting of TAMs: can we be more clever than cancer cells? Cell Mol. Immunol. 21 (12), 1376–1409. 10.1038/s41423-024-01232-z 39516356 PMC11607358

[B31] Lee G. S. Jeong H. Y. Yang H. G. Seo Y. R. Jung E. G. Lee Y. S. (2021). Astragaloside IV suppresses hepatic proliferation in regenerating rat liver after 70% partial hepatectomy *via* down-regulation of cell cycle pathway and DNA replication. Molecules 26 (10), 2895. 10.3390/molecules26102895 34068164 PMC8152973

[B32] Li J. Huang L. Wang S. Yao Y. Zhang Z. (2016). Astragaloside IV attenuates inflammatory reaction *via* activating immune function of regulatory T-cells inhibited by HMGB1 in mice. Pharm. Biol. 54 (12), 3217–3225. 10.1080/13880209.2016.1216133 27564970

[B33] Li S. Dou B. Shu S. Wei L. Zhu S. Ke Z. (2022a). Suppressing NK cells by astragaloside IV protects against acute ischemic stroke in mice *via* inhibiting STAT3. Front. Pharmacol. 12, 802047. 10.3389/fphar.2021.802047 PMC8852846 35185544

[B34] Li Q. Han J. Yang Y. Chen Y. (2022b). PD-1/PD-L1 checkpoint inhibitors in advanced hepatocellular carcinoma immunotherapy. Front. Immunol. 13, 1070961. 10.3389/fimmu.2022.1070961 PMC9806143 36601120

[B35] Li Z. Hu E. Zheng F. Wang S. Zhang W. Luo J. (2023). The effects of astragaloside IV on gut microbiota and serum metabolism in a mice model of intracerebral hemorrhage. Phytomedicine 121, 155086. 10.1016/j.phymed.2023.155086 37783132

[B36] Li M. Niu Y. Tian L. Zhang T. Zhou S. Wang L. (2024). Astragaloside IV alleviates macrophage senescence and d-galactose-induced bone loss in mice through STING/NF-κB pathway. Int. Immunopharmacol. 129, 111588. 10.1016/j.intimp.2024.111588 38290207

[B37] Li Z. Zhang C. Huang G. Zhang Z. Wang Q. Liu X. (2025). Deletion of Tfap2a in hepatocytes and macrophages promotes the progression of hepatocellular carcinoma by regulating SREBP1/FASN/ACC pathway and anti-inflammatory effect of IL10. Cell Death Dis. 16 (1), 245. 10.1038/s41419-025-07500-8 40180937 PMC11968862

[B38] Lian S. L. Lu Y. T. Lu Y. J. Yao Y. L. Wang X. L. Jiang R. Q. (2024). Tumor-associated macrophages promoting PD-L1 expression in infiltrating B cells through the CXCL12/CXCR4 axis in human hepatocellular carcinoma. Am. J. Cancer Res. 14 (2), 832–853. 10.62347/ZIAX8828 38455420 PMC10915331

[B39] Liang Y. Chen B. Liang D. Quan X. Gu R. Meng Z. (2023). Pharmacological effects of astragaloside IV: a review. Molecules 28 (16), 6118. 10.3390/molecules28166118 37630371 PMC10458270

[B40] Liu B. Zhao H. Wang Y. Zhang H. Ma Y. (2020). Astragaloside IV attenuates lipopolysaccharides-induced pulmonary epithelial cell injury through inhibiting autophagy. Pharmacology 105 (1-2), 90–101. 10.1159/000502865 31554002

[B41] Liu H. Luo S. Sha X. Chen Z. Yang D. (2023). Astragaloside IV inhibits pathological functions of gastric cancer-associated fibroblasts through regulation of the HOXA6/ZBTB12 axis. Acta Pharm. 73 (3), 423–439. 10.2478/acph-2023-0033 37708965

[B42] Liu X. Wang S. Wu X. Zhao Z. Jian C. Li M. (2024). Astragaloside IV alleviates depression in rats by modulating intestinal microbiota, T-Immune balance, and metabolome. J. Agric. Food Chem. 72 (1), 259–273. 10.1021/acs.jafc.3c04063 38064688

[B43] Liu F. Li X. Zhang Y. Ge S. Shi Z. Liu Q. (2025a). Targeting tumor-associated macrophages to overcome immune checkpoint inhibitor resistance in hepatocellular carcinoma. J. Exp. Clin. Cancer Res. 44 (1), 227. 10.1186/s13046-025-03490-9 PMC12323087 40764998

[B44] Liu Y. An T. Que Z. Luo B. Shi W. Zhou Y. (2025b). Inhalable herbal nano-liposomes Co-Delivering astragaloside IV and polyphyllin VII reprogram immunosuppressive microenvironment to overcome Anti-PD-1 resistance in NSCLC. Biol. Proced. Online 27 (1), 43. 10.1186/s12575-025-00302-4 PMC12604339 41214544

[B45] Loenarz C. Schofield C. J. (2011). Physiological and biochemical aspects of hydroxylations and demethylations catalyzed by human 2-oxoglutarate oxygenases. Trends Biochem. Sci. 36 (1), 7–18. 10.1016/j.tibs.2010.07.002 20728359

[B46] Luo Z. Wang Y. Xue M. Xia F. Zhu L. Li Y. (2021). Astragaloside IV ameliorates fat metabolism in the liver of ageing mice through targeting mitochondrial activity. J. Cell Mol. Med. 25 (18), 8863–8876. 10.1111/jcmm.16847 34402182 PMC8435431

[B47] Ma Y. Li Y. Wu T. Wang Q. (2023). Astragaloside IV attenuates programmed death-ligand 1-Mediated immunosuppression during liver cancer development *via* the miR-135b-5p/CNDP1 axis. Cancers (Basel) 15 (20), 5048. 10.3390/cancers15205048 37894415 PMC10605108

[B48] Mantovani A. Marchesi F. Malesci A. Laghi L. Allavena P. (2017). Tumour-associated macrophages as treatment targets in oncology. Nat. Rev. Clin. Oncol. 14, 399–416. 10.1038/nrclinonc.2016.217 28117416 PMC5480600

[B49] Mantovani A. Allavena P. Marchesi F. Garlanda C. (2022). Macrophages as tools and targets in cancer therapy. Nat. Rev. Drug Discov. 21 (11), 799–820. 10.1038/s41573-022-00520-5 35974096 PMC9380983

[B50] Min L. Wang H. Qi H. (2022). Astragaloside IV inhibits the progression of liver cancer by modulating macrophage polarization through the TLR4/NF-κB/STAT3 signaling pathway. Am. J. Transl. Res. 14 (3), 1551–1566. 35422920 PMC8991133

[B51] Murray P. J. Allen J. E. Biswas S. K. Fisher E. A. Gilroy D. W. Goerdt S. (2014). Macrophage activation and polarization: nomenclature and experimental guidelines. Immunity 41 (1), 14–20. 10.1016/j.immuni.2014.06.008 25035950 PMC4123412

[B52] Pan X. Zhu Q. Pan L. L. Sun J. (2022). Macrophage immunometabolism in inflammatory bowel diseases: from pathogenesis to therapy. Pharmacol. Ther. 238, 108176. 10.1016/j.pharmthera.2022.108176 35346728

[B53] Peng D. Fu M. Wang M. Wei Y. Wei X. (2022). Targeting TGF-β signal transduction for fibrosis and cancer therapy. Mol. Cancer 21 (1), 104. 10.1186/s12943-022-01569-x 35461253 PMC9033932

[B54] Pu Y. Ji Q. (2022). Tumor-associated macrophages regulate PD-1/PD-L1 immunosuppression. Front. Immunol. 13, 874589. 10.3389/fimmu.2022.874589 35592338 PMC9110638

[B55] Qin L. Wang Y. Liang Y. Li Q. Xie X. Zhang H. (2023). Astragaloside IV alleviates atorvastatin-induced hepatotoxicity *via* AMPK/SIRT1 pathway. Pharmacology 108 (1), 74–82. 10.1159/000527231 36423574

[B56] Ruan Z. Li Y. Chen Y. (2026). Astragaloside IV alleviates neuroinflammation in cerebral ischemia-reperfusion injury by inhibiting HTR2B-mediated microglial M1 polarization. Neurol. Res. 48 (2), 178–191. 10.1080/01616412.2025.2528158 40641386

[B57] Scott C. L. Zheng F. De Baetselier P. Martens L. Saeys Y. De Prijck S. (2016). Bone marrow-derived monocytes give rise to self-renewing and fully differentiated Kupffer cellsNature. Communications 7, 10321. 10.1038/ncomms10321 26813785 PMC4737801

[B58] Shim M. K. Song S. K. Jeon S. I. Hwang K. Y. Kim K. (2022). Nano-sized drug delivery systems to potentiate the immune checkpoint blockade therapy. Expert Opin. Drug Deliv. 19 (6), 641–652. 10.1080/17425247.2022.2081683 35603410

[B59] Shiri A. M. Zhang T. Bedke T. Zazara D. E. Zhao L. Lücke J. (2024). IL-10 dampens antitumor immunity and promotes liver metastasis *via* PD-L1 induction. J. Hepatol. 80 (4), 634–644. 10.1016/j.jhep.2023.12.015 38160941 PMC10964083

[B60] Su X. Yan X. Zhang H. (2025). The tumor microenvironment in hepatocellular carcinoma: mechanistic insights and therapeutic potential of traditional Chinese medicine. Mol. Cancer 24 (1), 173. 10.1186/s12943-025-02378-8 40495147 PMC12150456

[B61] Sun X. Cai W. Li H. Gao C. Ma X. Guo Y. (2025). Endothelial-like cancer-associated fibroblasts facilitate pancreatic cancer metastasis *via* vasculogenic mimicry and paracrine signalling. Gut 74 (9), 1437–1451. 10.1136/gutjnl-2024-333638 40122596

[B62] Tang W. Wang X. Han B. Jiang S. H. Cao H. (2025). Tumor-associated macrophages in cancer: from mechanisms to application. Mol. Biomed. 6 (1), 145. 10.1186/s43556-025-00396-y 41417432 PMC12717352

[B63] Tripathi A. Debelius J. Brenner D. A. Karin M. Loomba R. Schnabl B. (2018). The gut-liver axis and the intersection with the microbiomeNature reviews. Gastroenterology & Hepatology 15 (7), 397–411. 10.1038/s41575-018-0011-z 29748586 PMC6319369

[B64] Wang Z. Feng C. Chen G. Cai Q. (2025). Astragaloside IV regulates macrophage polarization *via* the TLR4/NF-κB/STAT3 pathway to inhibit the malignant phenotype of renal clear cell carcinoma. Naunyn Schmiedeb. Arch. Pharmacol. 398 (11), 15573–15586. 10.1007/s00210-025-04219-3 40343452

[B65] Wang R. Chen J. Ye F. Li Y. Chen T. Dai M. (2026). Astragaloside IV ameliorates allergic rhinitis comorbid with asthma by inhibiting the antigen-presenting function of B cells. Basic Clin. Pharmacol. Toxicol. 139 (1), e70247. 10.1111/bcpt.70247 42233266

[B66] Wellen K. E. Hatzivassiliou G. Sachdeva U. M. Bui T. V. Cross J. R. Thompson C. B. (2009). ATP-citrate lyase links cellular metabolism to histone acetylation. Science 324 (5930), 1076–1080. 10.1126/science.1164097 19461003 PMC2746744

[B67] Wu T. Wu S. Gao H. Liu H. Feng J. Yin G. (2024). Astragaloside IV augments anti-PD-1 therapy to suppress tumor growth in lung cancer by remodeling the tumor microenvironment. Eur. J. Histochem 68 (4), 4098. 10.4081/ejh.2024.4098 39440587 PMC11558310

[B68] Xu H. Jing-Bo W. Chen Y. P. Huang W. Wei Z. B. (2024). Astragaloside IV protects against IL-1β-Induced chondrocyte damage *via* activating autophagy. Curr. Mol. Med. 24 (11), 1382–1389. 10.2174/0115665240249154231016080115 37855351

[B69] Xu J. Ding L. Mei J. Hu Y. Kong X. Dai S. (2025). Dual roles and therapeutic targeting of tumor-associated macrophages in tumor microenvironments. Signal Transduct. Target Ther. 10 (1), 268. 10.1038/s41392-025-02325-5 40850976 PMC12375796

[B70] Xue Z. Liu J. Xing W. Mu F. Wu Y. Zhao J. (2024). Hypoxic glioma-derived exosomal miR-25-3p promotes macrophage M2 polarization by activating the PI3K-AKT-mTOR signaling pathway. J. Nanobiotechnology 22 (1), 628. 10.1186/s12951-024-02888-5 39407269 PMC11481566

[B71] Yang X. Wang F. (2019). The effect of astragaloside IV on JAK2-STAT6 signalling pathway in mouse model of ovalbumin-induced asthma. J. Anim. Physiol. Anim. Nutr. Berl. 103 (5), 1578–1584. 10.1111/jpn.13114 31148265

[B72] Yang Y. Guo J. Huang L. (2020). Tackling TAMs for cancer immunotherapy: it's nano time. Trends Pharmacol. Sci. 41 (10), 701–714. 10.1016/j.tips.2020.08.003 32946772 PMC7652091

[B73] Yang Y. Hong M. Lian W. W. Chen Z. (2022). Review of the pharmacological effects of astragaloside IV and its autophagic mechanism in association with inflammation. World J. Clin. Cases 10 (28), 10004–10016. 10.12998/wjcc.v10.i28.10004 36246793 PMC9561601

[B74] Yang Q. Q. Zhang H. Y. Duan X. H. Li M. H. Sun J. Tian L. X. (2024). Astragaloside IV targeting autophagy of T cells improves inflammation of asthma. J. Asian Nat. Prod. Res. 26 (6), 699–713. 10.1080/10286020.2023.2294069 38213072

[B75] Yang G. Chen J. Y. Gong C. A. Wang R. Han L. Wang J. (2025a). Astragaloside IV-loaded zeolitic imidazolate framework-8 based on supercritical fluid as anti-solvent technology to improve bioavailability and anti-tumor activity against non-small cell lung cancer. Cancer Cell Int. 25 (1), 386. 10.1186/s12935-025-04028-x PMC12584494 41188833

[B76] Yang J. Jiang H. Wu C. Lin Y. Tan G. Zhan J. (2025b). Copper silicate nanoparticle-mediated delivery of astragaloside-IV for osteoarthritis treatment by remodeling the articular cartilage microenvironment. J. Control Release 381, 113583. 10.1016/j.jconrel.2025.113583 40032006

[B77] Yang Y. Guo J. Ye G. Li N. (2025c). Astragalus Mongholicus for idiopathic pulmonary Fibrosis treatment: from molecules to systems. Curr. Mol. Med. 26, 687–698. 10.2174/0115665240396394250911235834 41031494

[B78] Yang Y. Zhang Y. Feng X. Gao F. Ma Y. Yang S. (2026a). Pleione bulbocodioides polysaccharides reprogram tumor-associated macrophages *via* JAK2/STAT3 inhibition to suppress hepatocellular carcinoma. J. Hepatocell. Carcinoma 13, 578276. 10.2147/JHC.S578276 PMC13037536 41924547

[B79] Yang J. Wang J. Zhang W. He H. Wang C. Zhang Y. (2026b). Astragaloside IV directly targets PPP1R14B to sensitize prostate cancer to anti-PD-1 immunotherapy by remodeling the CX3CL1/CD8+ T cell axis. Phytomedicine 155, 158158. 10.1016/j.phymed.2026.158158 41950731

[B80] Yue G. Wang C. Liu B. Wu M. Huang Y. Guo Y. (2020). Liposomes co-delivery system of doxorubicin and astragaloside IV co-modified by folate ligand and octa-arginine polypeptide for anti-breast cancer. RSC Adv. 10 (20), 11573–11581. 10.1039/c9ra09040a 35496626 PMC9050494

[B81] Zeng Y. Huang J. Duan T. Wang X. (2026). Astragaloside IV inhibits nasopharyngeal carcinoma progression by inhibiting SATB2/Wnt/PD-L1 pathway and enhancing the killing activity of T cells. Eur. J. Histochem 70 (2), 4540. 10.4081/ejh.2026.4540 42206678 PMC13273206

[B82] Zhang L. Li Z. Skrzypczynska K. M. (2026). Single-cell analyses inform mechanisms of myeloid-targeted therapies in Colon Cancer. Cell 181(2), 442–459.e29. 10.1016/j.cell.2020.03.048 32302573

[B83] Zhang W. D. Zhang C. Liu R. H. Li H. L. Zhang J. T. Mao C. (2006). Preclinical pharmacokinetics and tissue distribution of a natural cardioprotective agent astragaloside IV in rats and dogs. Life Sci. 79 (8), 808–815. 10.1016/j.lfs.2006.02.032 16564551

[B84] Zhang J. Wu C. Gao L. Du G. Qin X. (2020). Astragaloside IV derived from astragalus membranaceus: a research review on the pharmacological effects. Adv. Pharmacol. 87, 89–112. 10.1016/bs.apha.2019.08.002 32089240

[B85] Zhang L. Gao J. Li Z. Liu J. Zhang C. Liu J. (2023). Astragaloside IV relieves IL-1β-induced human nucleus pulposus cells degeneration through modulating PI3K/Akt signaling pathway. Med. Baltim. 102 (33), e34815. 10.1097/MD.0000000000034815 PMC10443759 37603510

[B86] Zhang X. Zhang F. Li Y. Fan N. Zhao K. Zhang A. (2024). Blockade of PI3K/AKT signaling pathway by Astragaloside IV attenuates ulcerative colitis *via* improving the intestinal epithelial barrier. J. Transl. Med. 22 (1), 406. 10.1186/s12967-024-05168-w 38689349 PMC11061986

[B87] Zhang S. Li S. Cui L. Xie S. Wang Y. (2025a). Astragaloside IV attenuates angiotensin II-Induced inflammatory responses in endothelial cells: involvement of Mitochondria. J. Inflamm. Res. 18, 3951–3967. 10.2147/JIR.S504427 PMC11927501 40125084

[B88] Zhang L. Xu W. Zeng Y. Wang L. Luo J. Zhou X. (2025b). Astragaloside IV accelerates hematopoietic reconstruction by improving the AMPK/PGC1α-mediated mitochondrial function in hematopoietic stem cells. Chin. Med. 20 (1), 44. 10.1186/s13020-025-01092-3 PMC11963557 40170084

[B89] Zheng C. Zheng L. Yoo J. K. Guo H. Zhang Y. Guo X. (2017). Landscape of infiltrating T cells in liver cancer revealed by single-cell sequencing. Cell 169 (7), 1342–1356.e16. 10.1016/j.cell.2017.05.035 28622514

[B90] Zhou B. Zhou D. L. Wei X. H. Zhong R. Y. Xu J. Sun L. (2017). Astragaloside IV attenuates free fatty acid-induced ER stress and lipid accumulation in hepatocytes *via* AMPK activation. Acta Pharmacol. Sin. 38 (7), 998–1008. 10.1038/aps.2016.175 28344322 PMC5519246

[B91] Zhou J. Li L. Pu Y. Li H. Wu X. Wang Z. (2024). Astragaloside IV inhibits colorectal cancer metastasis by reducing extracellular vesicles release and suppressing M2-type TAMs activation. Heliyon 10 (10), e31450. 10.1016/j.heliyon.2024.e31450 38831823 PMC11145472

[B92] Zhu Y. Zhou Z. Du X. Lin X. Liang Z. M. Chen S. (2025). Cancer cell-derived arginine fuels polyamine biosynthesis in tumor-associated macrophages to promote immune evasion. Cancer Cell 43 (6), 1045–1060.e7. 10.1016/j.ccell.2025.03.015 40185095

[B93] Zou X. Xiao H. Bai X. Zou Y. Hu W. Lin X. (2022). Astragaloside IV drug-loaded exosomes (AS-IV EXOs) derived from endothelial progenitor cells improve the viability and tube formation in high-glucose impaired human endothelial cells by promoting miR-214 expression. Endokrynol. Pol. 73 (2), 336–345. 10.5603/EP.a2022.0011 35593682

